# Research education and training for nurses and allied health professionals: a systematic scoping review

**DOI:** 10.1186/s12909-022-03406-7

**Published:** 2022-05-19

**Authors:** Olivia King, Emma West, Sarah Lee, Kristen Glenister, Claire Quilliam, Anna Wong Shee, Hannah Beks

**Affiliations:** 1 Western Alliance, 25 Ryot St, Warrnambool, 3280 VIC Australia; 2 Barwon Health, Geelong, VIC Australia; 3 Monash University, Clayton, VIC Australia; 4 Deakin University, Geelong, VIC Australia; 5 The University of Melbourne, Wangaratta and Shepparton, VIC Australia; 6 Grampians Health, Ballarat, VIC Australia

**Keywords:** Research education, Research capacity building, Evidence-based practice, Health settings

## Abstract

**Background:**

Research capacity building (RCB) initiatives have gained steady momentum in health settings across the globe to reduce the gap between research evidence and health practice and policy. RCB strategies are typically multidimensional, comprising several initiatives targeted at different levels within health organisations. Research education and training is a mainstay strategy targeted at the individual level and yet, the evidence for research education in health settings is unclear. This review scopes the literature on research education programs for nurses and allied health professionals, delivered and evaluated in healthcare settings in high-income countries.

**Methods:**

The review was conducted systematically in accordance with the Joanna Briggs Institute scoping review methodology. Eleven academic databases and numerous grey literature platforms were searched. Data were extracted from the included full texts in accordance with the aims of the scoping review. A narrative approach was used to synthesise findings. Program characteristics, approaches to program evaluation and the outcomes reported were extracted and summarised.

**Results:**

Database searches for peer-reviewed and grey literature yielded 12,457 unique records. Following abstract and title screening, 207 full texts were reviewed. Of these, 60 records were included. Nine additional records were identified on forward and backward citation searching for the included records, resulting in a total of 69 papers describing 68 research education programs.

Research education programs were implemented in fourteen different high-income countries over five decades. Programs were multifaceted, often encompassed experiential learning, with half including a mentoring component. Outcome measures largely reflected lower levels of Barr and colleagues’ modified Kirkpatrick educational outcomes typology (e.g., satisfaction, improved research knowledge and confidence), with few evaluated objectively using traditional research milestones (e.g., protocol completion, manuscript preparation, poster, conference presentation). Few programs were evaluated using organisational and practice outcomes. Overall, evaluation methods were poorly described.

**Conclusion:**

Research education remains a key strategy to build research capacity for nurses and allied health professionals working in healthcare settings. Evaluation of research education programs needs to be rigorous and, although targeted at the individual, must consider longer-term and broader organisation-level outcomes and impacts. Examining this is critical to improving clinician-led health research and the translation of research into clinical practice.

**Supplementary Information:**

The online version contains supplementary material available at 10.1186/s12909-022-03406-7.

## Introduction

The translation of research evidence into health practice and policy relies on healthcare organisations and systems having sufficient research capacity and capability [1–3]. Health organisation executives and policymakers globally, recognise the need to invest in research capacity building (RCB) initiatives and interventions that are delivered in healthcare settings [2–4]. RCB strategies encompass a range of initiatives designed to promote individual, team and organisation research skills, competence and to influence attitudes towards research [2, 5–7]. Initiatives designed to build individual and organisational research capacity may include education and training programs, funding for embedded researchers (e.g., fellowships, scholarships) and other research support roles (e.g., research librarians, knowledge-brokers), strategic collaborations with academic partners and developing research infrastructure [2, 6, 8]. RCB strategies often comprise a combination of the aforementioned approaches [8] and notably, research education and training programs are a sustaining feature of many [2, 3, 6, 8–11]. This is likely related to the insufficient coverage of research in undergraduate health curricula and the need for supplementary education to fill research knowledge and skill gaps, particularly for non-medically trained healthcare professionals. Medically trained healthcare professionals typically have a greater inclination toward and engagement in research than their nurse and allied health counterparts [4, 8, 12, 13]. Given that nursing and allied health form the majority of the health workforce [14, 15], there is increasing interest in RCB strategies that target nurses and allied health professionals to enhance the delivery of evidence-informed care across all healthcare settings and services [8, 16–18]. Allied health comprises a range of autonomous healthcare professions including physiotherapy, social work, podiatry, and occupational therapy [16].

This review was commissioned by an academic health science centre in Australia, to inform the research education and training component of its health organisation RCB strategy. Given the typically multidimensional nature of RCB strategies, their functions and impacts at the various levels are inextricably related [2, 5]. This makes the discernment between research education and training interventions and other elements of strategies a fraught endeavour. For example, embedded researchers may form part of a broader organisational RCB strategy, and in the scope of their work, may perform an ad hoc education function (e.g., through their interactions with novice researchers) [11, 19]. Aligning with the purpose of this work, this review defines research education and training programs as organised initiatives or interventions that are either discrete (e.g., standalone workshops or research days) or longer in their duration (e.g., research courses or a series of workshops or lectures) wherein curriculum is developed and shared with multiple individuals or participants, with a view to develop and apply research skills [2, 5]. Healthcare settings are considered those wherein the provision of healthcare is considered core business (e.g., hospitals, community-based health services, cancer care services, family medicine clinics) and is therefore the setting in which research evidence needs to be applied or translated to reduce the gap between research knowledge and practice [2, 20].

An initial search of Cochrane Database of Systematic Reviews, Joanna Briggs Institute’s Evidence Synthesis, PROSPERO, and Google Scholar for reviews of research education and training programs delivered in health settings, yielded no existing or planned reviews. On further cursory review of the RCB and research education literature, and concomitant discussions with four content experts (i.e., educators, academic and clinician researchers concerned with research capacity building), it became apparent that research education programs take different forms, occur in pockets within health organisations across health districts and regions, are not always formally evaluated, and often fail to account for adult learning principles and theories. The decision to conduct a scoping review, rather than a conventional systematic review, was based on three key factors: 1) the heterogeneity evident in research education program characteristics; 2) the absence of an existing synthesis of evidence for research education programs delivered in health settings [5]; and 3) the need to identify the gaps in knowledge about these programs.

This systematic scoping review sought to scope the research education and training programs delivered to nurses and allied health professionals working in health settings and the evidence supporting these approaches. The specific review objectives were to describe the:

1. Types of research education programs delivered in health settings in high-income countries
2. Theoretical or pedagogical principles that underly the programs
3. Approaches to research education program evaluation
4. Types of outcomes reported

## Methods

This review used the Joanna Briggs Institute’s (JBI) scoping review methodology. As per the JBI methodology, search terms were developed for Population, Concept and Context (PCC). The review question, objectives, inclusion/exclusion criteria and search strategies were developed and documented in advance (Additional File 1 Scoping Review Protocol). The review is reported in accordance with the Preferred Reporting Items for Systematic reviews and Meta-Analyses (PRISMA) extension for scoping reviews (Additional File 2 PRISMA-ScR checklist [21]).

### Search strategy

The researchers identified a set of key papers based on their knowledge of contemporary research education programs and in consultation with four content experts from two high-income countries. They used these papers to identify the key search terms. In consultation with the research librarians (SH and HS, see acknowledgements), the research team conducted preliminary scoping searches to test the search terms and strategy (between 3 March – 10 March 2022). These searches informed decisions about final search terms. A tailored search strategy was developed for each academic database (Additional file 3 Search Strategy).

Academic databases searched included PubMed, Ovid MEDLINE, Embase, CINAHL, VOCEDPlus, PEDro, Scopus, ERIC, Informit Health Database, JBI, and Google Scholar. Selected grey literature platforms as determined by our knowledge of relevant websites and organisations, were searched. Where larger search yields were observed (e.g., via Google and Google Scholar), the first 250 items were reviewed, only (Additional file 4 Grey literature search). The final research database searches were conducted between 12 and 15 March 2022 by a researcher with extensive systematic literature searching experience (Author 2) in consultation with a research librarian. Grey literature searches were conducted on 17 March 2022. Searches of the reference lists of included records and forward citation searches were undertaken.

### Inclusion criteria and exclusion criteria

Literature was selected according to defined inclusion and exclusion criteria developed using the PCC framework (see Table 1). Research education or capacity building programs delivered to qualified health professionals, working in health settings (excluding programs delivered as part of tertiary study) in high-income countries (HIC) as defined by the Organisation for Economic Co-operation and Development (OECD), were included [22]. The decision to include studies published in HICs only was made with a view to introduce a level of homogeneity around the broader resource contexts of the study populations [23, 24]. No date limits applied, and all types of literature published up to 17 March 2022 were included. Literature published in English only was included, due to resource limitations.

**Table 1:** Inclusion and exclusion criteria

	Inclusion criteria	Exclusion criteria
**Population**	Health professionals working in healthcare settings including nurses, midwives, allied health professionals (e.g., physiotherapists, dietitians, speech pathologists, social workers, occupational therapists, podiatrists, dietitians), pharmacists^a^	Medical doctors only Health professionals working in non-health / academic settings Undergraduate students
**Concept**	Research capacity building/ development programs, research-orientated continuing education, in-services, training, workshops, workplace learning or mentorship Evaluated programs^b^	Fellowships, scholarships, or other new roles (e.g., knowledge brokers, embedded researchers, librarian), research education as part of tertiary course, global research capacity building frameworks and programs
**Context**	Research or capacity building programs delivered in the healthcare setting in high-income countries (according to OECD criteria) [22]	Programs delivered in academic institutions and non-health settings or in low and middle-income countries

^a^ Health professionals were not limited to those that are accredited or registered, but rather included any health worker that was situated in a healthcare setting

^b^ Evaluation was considered if there was an informal or formal approach to measuring and describing the outcomes and/or impacts of the program, to determine whether it met its objectives

### Study selection, quality appraisal and data extraction

Citations were imported into Covidence (Veritas Health Innovation, Melbourne, Australia) for screening. Titles and abstracts were independently screened by two reviewers initially, with conflicts resolved by a third (independent) reviewer. Similarly, full texts were reviewed by two researchers and the reasons for exclusion were noted (Additional file 5 Excluded studies). Data was extracted from the included texts by five researchers. Formal quality appraisal is not typically undertaken as part of scoping review methodology and was not undertaken for the papers included in this review [25].

Data extracted were tabulated and results were synthesized using a descriptive approach guided by the review objectives as per a scoping review methodology. Outcomes measured and reported in the papers were mapped to the modified Kirkpatrick’s educational outcomes typology [26, 27]. Recognising the complex interactions between individuals, research education programs, organisational and other factors, and the various outcomes produced [2], the modified Kirkpatrick’s typology gives rise to the identification of outcome measures at multiple levels or within these inter-related domains [26].

## Results

**Fig. 1:**
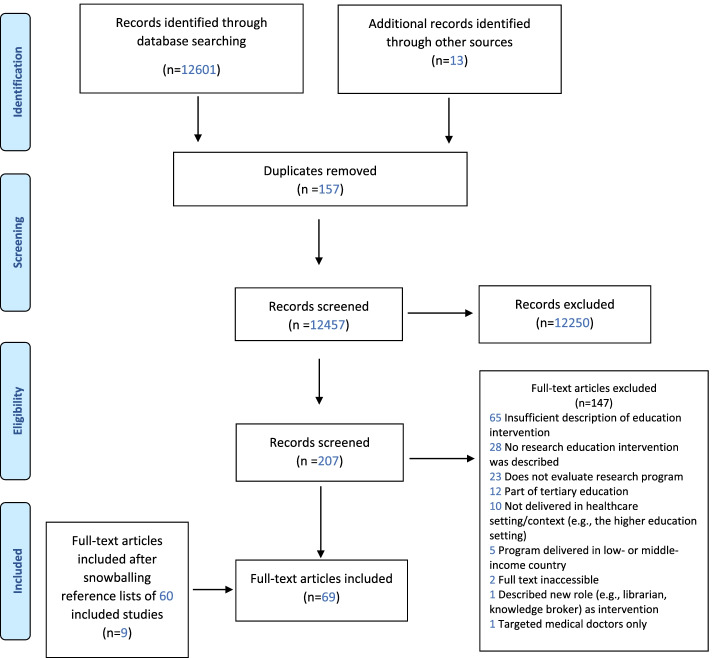
PRISMA Flow Diagram

Of the 207 citations considered for full text screening, 60 met the inclusion criteria and nine additional papers were located through a citation search of the initial set (Fig. 1 PRISMA Flow Diagram) [28].

### Research education program characteristics

#### When, where and to whom research education programs were delivered

**Fig. 2:**
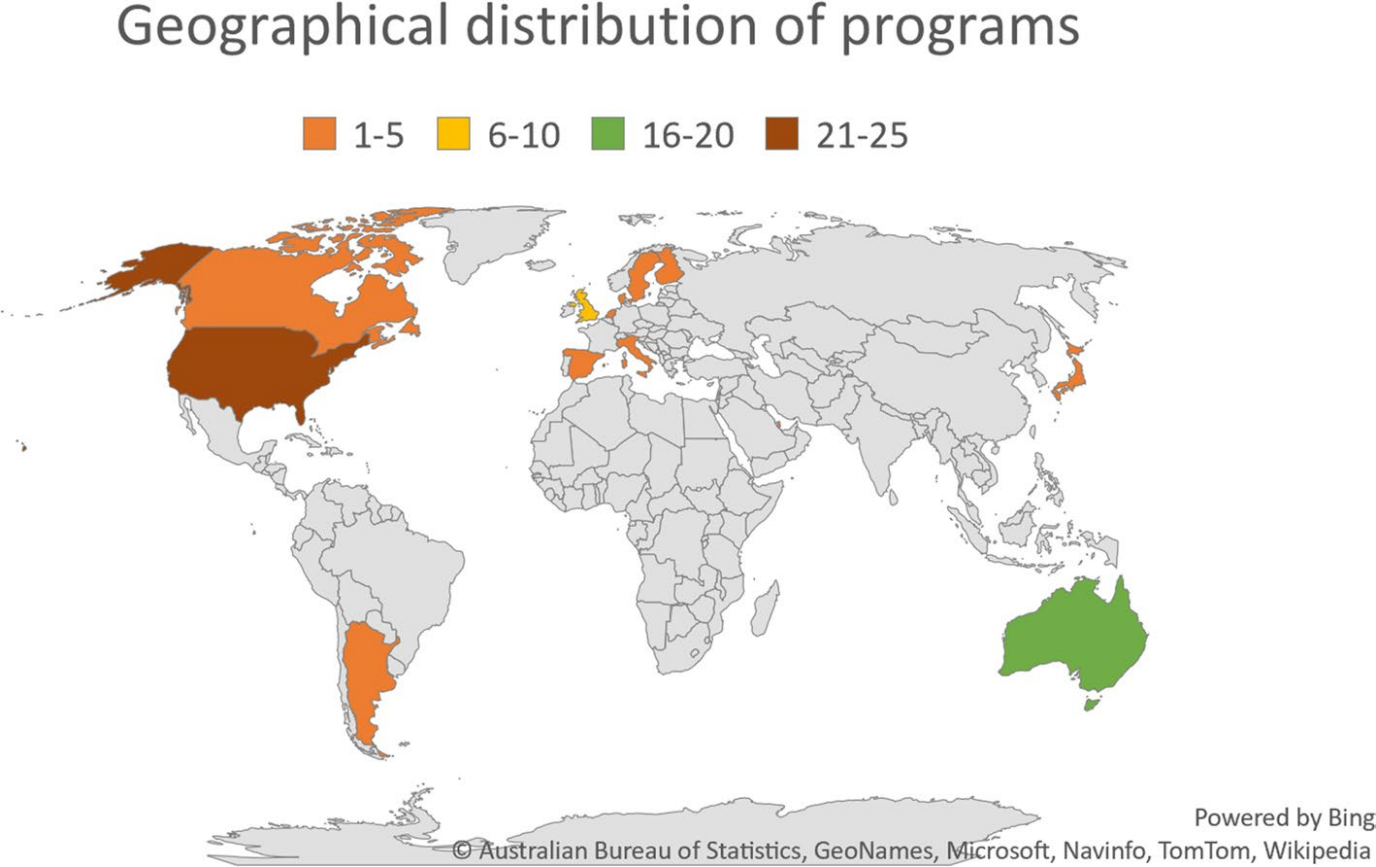
Geographical distribution of research education programs. This image was generated by the authors via Microsoft Excel using the Map function

A total of 69 papers, describing 68 research education and training programs were reviewed. The implementation of the programs spanned five decades, with almost half (*n =* 33) implemented in the most recent decade. Research education programs were delivered in the United States of America (*n =* 22), Australia (*n =* 20), the United Kingdom (*n =* 9), Canada (*n =* 5), Denmark (*n =* 2), Qatar (*n =* 2), and one each in Argentina, Finland, Japan, Italy, Singapore, Sweden, Spain, and The Netherlands. The geographical distribution of programs by country is presented in Fig. 2. Research education programs were targeted and delivered to different healthcare professional groups. Programs were delivered most frequently to nurses and midwives (*n =* 35), then mixed professional groups (*n =* 18), allied health (*n =* 13), and pharmacists (*n =* 2). The characteristics of included programs are provided in Table 2.

**Table 2:** Research education program characteristics

Citation	Program, country, year(s) of implementation	Education program description, curriculum, and aims	Number of participants, profession/s, setting	Pedagogical tools and learning theories
**Program delivered before 1991 (*n =*** **1)**				
Warren & Heermann, 1998 [94]	Research Nurse Internship, USA, 1989–1998	Program description: 2-year program with 2 distinct phases: year 1 focuses on learning about research utilisation, year 2 focuses on conducting research utilisation projects Formal, yet highly interactive classes are held monthly and last between 90 minutes and full day in duration Participants are released from direct patient care by their manager, who is also responsible for creating a supportive learning and innovative environment Clinical nurse researchers coordinate the program; one-to-one mentoring provided by advanced practice nurses At the end of year 2, participants share their findings and recommendations within and beyond the organisation Curriculum: Year 1: research process, introduction to research critical appraisal, orientation to library resources, conducting a computer-based literature search, approaches to facilitate research dissemination and utilization Year 2: determining the impact and feasibility of the innovation, preparation and communication, project management, data analysis, dissemination of the results, and implementing change Aim: To facilitate the integration of research into clinical practice, support the professional development of clinical nurses and promote collaboration among clinicians (nurses and others), educators, and researchers	127 (of which 88 completed both years of the internship) Nurses 1 university medical centre	Collaborative learning Didactic learning Experiential learning Mentoring
**Program delivered between 1991 and 2000 (*n =*** **9)**				
Adamsen, Larsen, Bjerregaard, & Madsen, 2003 [77]	Basic Research Methodology for Nurses, Denmark, 1999–2000	Program description: Year-long, course delivered for one day (8 hours) per month by PhD level teachers that are active in research Course comprised a total of 19 days of classroom work, 120 hours of lectures, 2400 pages of required reading and practical exercises. Participants were also required to prepare a project proposal (protocol) Minimum of 3 hours guidance was provided to each participant by a PhD-qualified course teacher Curriculum: Basic research methodology and critical scientific reflection, (e.g., interview techniques, presentation skills, defending proposal) Aim: To enable students to plan a research project with a view to implementing the findings in clinical practice	37 Nurses 10 hospitals	Critical and problem-orientated pedagogy Didactic learning Experiential learning Mentoring
Bott, 2000 [29]	Critical Appraisal of Research Workshop, Canada, 1998	Program description: Single 5-hour interactive workshop comprising interactive lectures, reading, and practical learning within a large and a small group setting facilitated by a nephrology nurse who was skilled in research appraisal Curriculum: Critical appraisal of research and utilization of research in the practice setting Aim: To develop nurses’ critical appraisal skills and provide tools for nurse facilitators to upskill a broader nursing audience	30 Nurses Nephrology centres	Collaborative learning Didactic learning Experiential learning
Gething, Leelarthaepin, Burr, & Sommerville, 2001 [35]	Research Training Workshops, Australia, 1997–2000	Program description: Research training workshops delivered as part of a comprehensive RCB strategy Workshops varied in duration from 0.5–2 days and were delivered onsite by highly experienced and active researchers who were also available for follow-up consultation Neophyte researchers were linked with more experienced researchers for mentorship and consultancy Separate training program was offered to neophyte researchers aspiring to apply for grant funding Curriculum: Range of research topics covered including research design, implementation, analysis, dissemination, critiquing research findings, computer skills, and specialised topics. Aims: Education component of the strategy aimed to meet the needs of nurse researchers at different stages of participation in research Grant funding education component aimed to build research via an introduction to funded research	320 Nurses 1 large metropolitan regional health service	Didactic learning Mentoring
Hicks, 1994 [30]	Critical Research Reading Skills Study Day, England, year(s) of implementation not specified	Program description: Research study day comprising two 2-hour lectures, supportive written materials, and an activity in which participants appraised a research article at the start of the day and again at the end of the day with the lecturer Curriculum: Basic research methods and guidelines for appraising research Aims: To improve participants’ ability to read and critically appraise research as a precursor to translating research into practice	19 Midwives Hospital and community settings	Didactic learnin Experiential learning
Hundley, Milne, Leighton-Beck, Graham, & Fitzmaurice, 2000 [36]	Raising Research Awareness Among Midwives and Nurses, Scotland, 1997	Program description: Multifaceted intervention including research critical appraisal workshops, seminars, discussion and interest groups, and access to research infrastructure Research education and training program, implemented with and without concurrent policy and practice interventions Curriculum: Critical appraisal of research, research proposal development Aim: To increase research awareness among midwives and nurses	333 Nurses and midwives 1 acute NHS Trust	Collaborative learning Didactic learning Experiential learning
Kajermo, Nordström, Krusebrant, & Lützén, 2001 [88]	Dissemination and Utilization of Research Findings in Clinical Nursing Practice, Sweden, year(s) of implementation not specified	Program description: 2-year long part-time course comprising formal monthly classes lasting about 2.5 hours and experiential activities delivered by nurse researchers Managers of involved nurses overtly supported participation and approved the allocation of half day per week to research Curriculum: Research process, literature searches, research methods, reviewing and critical evaluation of research articles, developing a poster, relationship between research and quality improvement and the process of change Aim: To facilitate the dissemination and implementation of research findings into clinical practice	10 Nurses 2 acute-care teaching hospitals and 1 nursing home	Didactic learning Experiential learning
Mulhall, May, & Alexander, 2000 [41]	Research Utilization Workshops, UK, year(s) of implementation not specified	Program description: 2 blocks of 2-day workshops (4 in total) held 4–5 weeks apart Curriculum: Introduction to research, literature searching, and synthesis, research design, critiquing literature, research dissemination and implementation, theory of practice changes, evaluating practice change Aim: To help participants critically appraise research and implement research in their workplace	206 Nurses, mental health nurses, and midwives	Collaborative learning Didactic learning Experiential learning
O’Halloran, Pollock, Gottlieb, & Schwartz, 1996 [31]	Research Workshop, USA, year(s) of implementation not specified	Program description: Single full-day workshop developed and implemented by a Nursing Research Committee Followed semi structured format and included formal presentations and practical activities using a delphi research process to increase research knowledge and skills Support was provided by Nursing Research Committee members Curriculum: Formal presentations on experiential learning, Delphi studies, and writing research proposals. Experiential learning activity focused on the research process Aim: To provide nurses with a positive research experience, with a view to increase research self-efficacy, with an expectation this would increase nurses’ research activity	33 Nurses 1 large healthcare facility	Collaborative learning Didactic learning Experiential learning Self-efficacy theory
Rutledge, Mooney, Grant, & Eaton, 2004 [34]	Research Utilization Course, USA, 1997–2001	Program description: 1-day course targeted nurses with an intent to make evidence-based changes to nursing cancer care, as demonstrated by a proposal Didactic delivery of content by faculty experts in research utilisation and a discussion about the strengths and weakness of proposals Letter of support from manager required for each participant Curriculum: Research utilisation models, protocol development, literature critique methods Aim: To expand the scientific foundation in cancer nursing care	22 Nurses Multiple health services	Collaborative learning Didactic learning Experiential learning Mentoring
**Program delivered between 2001 and 2010 (*n =*** **25)**				
Allen, Boase, Piggott, et al., 2010 [42]	Practice Nurse Clinical Research Workshops, England, 2008–2009	Program description: 1 full and 4 half day stand-alone workshops were delivered over 4-month period A resource file which contained a set of convenient reference guides; scenario discussions, quizzes, and role play were developed and used to reinforced participants’ learning Workshop content was developed and delivered by research nurses, GPs, other researchers, data managers, and research administrators Curriculum: Nurse researcher role, participant recruitment and consent, following research protocols, data management and record keeping, common research issues, research context Aim: To increase nurses’ knowledge of clinical research and confidence to conduct research, promote the value of clinical research and the work of the cancer service, and promote further training opportunities	90 Nurses 67 general practices	Collaborative learning Didactic learning Experiential learning Quizzes
Bamberg, Perlesz, McKenzie & Read, 2010 [37]	Research and Evaluation Capacity-building Program in the Community Health Sector, Australia, 2008–2010	Program description: Research and evaluation training program delivered (five 2-hour sessions) as part of a broader RCB program Curriculum: Overview of evaluation, literature searches, qualitative research methods, designing questionnaires, and writing for publication Aim: To enhance research and evaluation capacity within the organisation	n not stated Mixed 1 large community-based health service	Ely’s Conditions for Change Model Mentoring Roger’s Diffusion of Innovation Theory
Corchon, Portillo, Watson, & Saracibar, 2011 [38]	Research Courses and Journal Clubs, Spain, 2007	Program description: A mentors’ network and an educational program consisting of research courses and journal clubs Mentors’ network comprised of nurses with MSc or PhD, who provided continuous support and research advice to participants Research course included a handbook with course contents, bibliography, and relevant articles Curriculum: Research course focused on literature searching and appraisal skills and the relevance of translating research evidence into practice Aim: To increase nursing research activity by enhancing research capability: knowledge, attitudes, and interest	89 Nurses 1 specialised teaching hospital	Collaborative learning Didactic learning
Demirdjian, Rodríguez, Vassallo, Irazola, & Rodríguez, 2017 [78]	Intensive Research and Management Capacity-building Program for Hospital Health Care Professionals, Argentina, 2006–2013	Program description: Annual 250-hour intensive course featuring research and management jointly organized by a hospital and university Course combined in-class activities (weekly 3-hr lectures and workshops) with offsite activities (integrative exercises, self-assessment questionnaires, discussion forums) delivered through online platform. Participants also required to develop a research project with with mentor supervision Curriculum: Block 1 (Research) included epidemiology, methodology, bibliographic search and evidence-based medicine, biostatistics. Block 2 (Management) included strategic planning, management projects and programs, health services research, quality improvement, health economics Aim: To provide paediatric professionals with practical tools to conduct research and management activities. Course aimed to demystify the publishing process and encourage participants to publish their work	295 Mixed paediatric professionals Hospital (mainly) and external public and private institutions	Didactic learning Collaborative learning Experiential learning Mentoring
Doyle & Harvey, 2005 [33]	Publishing Short Course, USA, 2003	Program description: Single session course, approximately 1.5–2 hours duration comprising informal lecture allowing for group discussion and question/answer, PowerPoint slides and handouts (relevant articles and tip sheets) Curriculum Identifying publishable research idea, what and where to publish, authorship, organizing and structuring a paper, submitting a paper, alternative dissemination methods, writing tools/resources Aim: To encourage participants to consider publishing the results of their work and to demystify the publishing process	40 Mixed 2 healthcare organisations	Collaborative learning Didactic learning
Duffy, Thomson, Hobbs, Niemeyer-Hackett, & Elpers, 2011 [87]	Leadership Journal Club, USA, 2010	Program description: Journal club held monthly at 12 pm, for 12 months. Led by Chief Nursing Officer (hospital) and Professor (nursing school) Research topics (related to nurse leadership) chosen by leads in discussion with participants and article sent via email a week before journal club Curriculum: Critical appraisal process, specific statistical methods or methodological issues, reflection on application of the results for practice and policy Aim: To increase awareness of nursing leadership research, develop leadership, competence in research appraisal, provide a forum to discuss ideas for practice changes, influence leadership decision-making	27 Nurse leaders 1 hospital	Collaborative learning
Harding, Stephens, Taylor, Chu, & Wilby, 2010 [46]	12-Week Allied Health Research Training Scheme, Australia, year(s) of implementation not specified	Program description: Mentored program to support allied health professionals to complete a systematic review. Comprising five 3-hour group workshops spaced over 12 weeks Candidates required written support from manager to participate and be released from clinical duties Curriculum: Formulating a research question, identifying literature, critical evaluation of papers, synthesising data (qualitative and quantitative methods) and writing the review Aim: To introduce research, whilst embedding a research culture within every day clinical practice	6 Allied health professionals 1 metropolitan health service	Collaborative learning Didactic learning Experiential learning Mentoring
Hart, Eaton, Buckner, et al., 2008 [55]	Computer-Based Learning EBP Program, USA, 2006	Program description: 3-month EBP-education program consisting of 3 computer-based learning modules designed to enhance EBP skills; each took 15–20 minutes to complete Curriculum: Refining the clinical question, how to read and understand a research paper, and utilizing research in clinical practice Aim: To improve participants’ EBP knowledge, attitudes, and skills	314 Nurses Five hospitals, physician practice groups, and outpatient services	Self-guided computer-based learning
Holden, Pager, Golenko, Ware, & Weare, 2012 [79]	Designated Research Team, Australia, 2009–2010	Program description: 15-month research capacity building intervention. Expressions of interest required multidisciplinary teams to have manager approval, a research idea, and at least one person in the team with some research experience Training was provided in alignment with research milestones (and recorded for access by researchers/teams unable to attend in person) Ongoing mentoring and protected time for one research team member (one day/week) and access to research resources were offered as part of the intervention Curriculum: Developing a research idea/research proposal, ethics applications, grant funding, qualitative and quantitative methods Aim: To develop research capacity by taking a team-based approach	37 Mixed 1 public health district	Collaborative learning Didactic learning Experiential learning Mentoring
Jansen & Hoeijmakers, 2013 [91]	Masterclass on Scientific Research Training for Public Health Professionals, Netherlands, 2008–2009	Program description: 18-month Masterclass divided into six 1-week long courses Different time intervals between the courses to apply learnings in practice, with a total time investment of 660 hours Access to university library and a university-based supervisor Curriculum: Identifying an operational problem, developing a research question, introduction to qualitative and quantitative methods, developing a research proposal, data analysis, writing a manuscript, implementation in practice and policy Aim: To train public health professionals to design and conduct practice or policy-related research	21 Mixed Multiple institutions	Didactic learning Experiential learning Mentoring
Land, Ward, & Taylor, 2002 [40]	Critical Appraisal Module, England, year(s) of implementation not specified	Program description: 7 EBP introductory modules were delivered over a 6-week period Workshops were 3-hours long and comprised introductory material and practical break-out group work Academics and practice development nurses facilitated the workshops, which were held at 2 NHS hospital sites to maximise attendance Curriculum: Introduction to critical appraisal, research questions, literature review, critical appraisal, implementing change Aim: To develop staff confidence in locating, appraising, and applying literature to practice; understanding of clinical audit measures, and demonstrate value of interdisciplinary team work	45 Mixed 1 NHS trust	Collaborative learning Didactic learning Experiential learning
Latimer & Kimbell, 2010 [73]	QMC Nursing Research Fellowship, USA, year(s) of implementation not specified	Program description: 8-month long competitive fellowship program with an education component Monthly research education sessions comprising didactic, experiential and peer-to-peer learning and group discussions, as well as self-guided learning via a textbook and homework between sessions Support provided by Masters and PhD-qualified nurses Curriculum: Fundamentals of research including ethics, research proposal development, literature searching, developing a research question, introduction to qualitative and quantitative methods, and data collection Aim: To educate nurses on research processes and for nurses to develop research or administrative funding proposals	10 Nurses 1 private hospital	Collaborative learning Didactic learning Experiential learning Mentoring
Levin, Fineout-Overholt, Melnyk, Barnes, & Vetter, 2011 [100]	Advancing Research and Clinical practice through close Collaboration (ARCC) Model, USA, year(s) of implementation not specified	Program description: 16-week program (intervention group) comprising a 4-week training program (intervention and control groups) followed by formal EBP mentoring for 12 weeks (intervention group only) Curriculum: Weekly 1-hour long classes covered EBP topics including: introduction to EBP, developing clinical questions, searching for evidence, critical appraisal Aim: To improve nurse participants’ beliefs about and implementation of EBP, group cohesion, job satisfaction, productivity, and nurse retention	46 Nurses 3 Home Health Care Program sites	ARCC (based on cognitive-behavioural theory and control theory) Didactic learning Experiential learning Mentoring
Mathers, Abel, & Chesson, 2004 [57]	Radiography Research Course, Scotland, year(s) of implementation not specified	Program description: 4 workshops held in two blocks over a 2-month period comprising 30 hours of course content and 1 independent study day Resource pack complemented the workshop delivery An experienced radiographer-researcher developed workshops and arranged guest lecturers including local health specialists, clinical directors, ethics committee members, librarians Curriculum: Introduction to clinical governance, research, audits, local research context, research in radiography, literature searching, critical appraisal skills, ethics and informed consent, research design and methods, coding and interpretation, report and article writing and submission, dissemination, and research presentations Resource pack included material on clinical governance strategies, critical appraisal, research methods, dissemination and reading recommendations Aim: To increase staff knowledge and implementation of clinical governance by developing their research appraisal, project planning and performance skills and their systematic practice review skills	12 Radiographers (Allied health) 1 NHS Trust	Didactic learning Experiential learning
McCluskey & Lovarini, 2005 [71]	Evidence-Based Practice Workshop, Australia, 2002	Program description: 2-day workshop held on weekends, with content delivered by a clinician-researchers and health librarian Follow-up support was provided by a clinician-researcher via email and telephone for 11 months Curriculum: EBP process, developing a researchable question, database searches, critical appraisal of quantitative and qualitative research, interpreting statistics, and implementing EBP Aim: To promote EBP knowledge and skills and equip participants with the skills overcome challenges to implementing EBP	106 Occupational therapists (Allied health) Multiple health services	Didactic learning Experiential learning Mentoring Roger’s Diffusion of Innovation theory Social cognitive theory
Milne, Krishnasamy, Johnston, & Aranda, 2007 [58]	Clinical Research Fellowship Programme, Australia, 2002	Program description: 12-week program comprised a journal club, with written materials and facilitator support when required for manuscript writing beyond the formal program period Facilitated by nurses with extensive clinical and research and EBP experience Program restricted to 10 participants to allow for sufficient support; all participants required manager support to participate and for potential changes arising from the project to be implemented Curriculum: Literature search and appraisal, research question and recommendation development, identify organisational culture barriers to evidence utilisation and strategies for change management Aim: To improve health professionals’ research utilization, written and oral research dissemination skills, and address research utilisation barriers	15 (over two programs) Mixed 1 health service	Collaborative learning Didactic learning Experiential learning
Murphy, Kalpakjian, Mullan, & Clauw, 2010 [59]	Practice-Oriented Research Training (PORT) Program, USA, year(s) of implementation not specified	Program description: 2-phase program of unspecified duration was facilitated by two health academics and academic expert guest speakers Phase 1 consisted of three 2-hour sessions offered weekly after regular work hours, phase 2 involved nine 1-hour sessions offered weekly Program involved seminars, independent work via a web-based platform and mentor support beyond the program until project completion; small cost to participants to cover textbook and food Curriculum: Discipline relevant examples on the fundamentals of clinical research, literature review, developing research questions, evaluating evidence, grant writing, research design, ethics and research proposals, statistics, and psychometrics Aim: To improve participants’ clinical research skills to promote the formulation and submission of translation-focused clinical research grants	38 Allied health 1 health service	Collaborative learning Didactic learning Experiential learning
Pennington, Roddam, Burton, Russell, & Russell, 2005 [60]	Speech and Language Therapy Research Training Program, England, 2001–2002	Program description: Training comprised 2 strategies: Strategy A involved 2.5 training days over 7 weeks and implementation of a guideline recommendation; Strategy B involved 5 training days, once a fortnight over 3 months and implementation of a guideline recommendation 2 speech and communication academics delivered the training where written practice guidelines were provided Participants kept diaries of rollout activities Curriculum: Strategy A included clinical governance, evidence-based health care, systematic review critical appraisal, randomized controlled trials, evidence-based guidelines and cohort and quasi-experimental studies Strategy B included content from strategy A and additional content based on Roger’s diffusion of innovation model Aim: To improve participants’ ability to introduce and manage evidence-based changes to clinical practice within their department	34 Speech and language therapists (Allied health) 17 departments across 1 NHS area	Collaborative learning Didactic learning Experiential learning Roger’s Diffusion of Innovation
Richardson & Carrick-Sen, 2011 [74]	Writing for Publication Programme, England, 2007–2009	Program description: 8-month long program, with monthly structured sessions of between 2- and 5-hours duration Sessions were delivered by experienced academics Curriculum: Introduction to academic writing, submission, the peer review process, defining the topic, choosing a journal, author guidelines, literature searching, writing style, referencing, and bibliographic software Aim: To encourage and support nurses to write a paper for a peer-reviewed journal	50 Nurses and midwives 1 NHS Trust	Collaborative learning Didactic learning Experiential learning Mentoring
Shatzer, Wolf, Hravnak, et al., 2010 [47]	Bedside to Byline, USA, year(s) of implementation not specified	Program description: 10-week program including didactic content, two 4-hour workshops and three structured 1:1 mentoring sessions with the workshop facilitator Participants developed a manuscript draft for journal publication or other type of publication Curriculum: Didactic content covered a range of elements across the publication process continuum Aim: To reduce barriers to nurses publishing and to increase participants’ self-efficacy related to scholarly writing	11 Nurses 2 community teaching hospitals	Collaborative learning Didactic learning Self-efficacy theory Mentoring
Swenson-Britt & Reineck, 2009 [61]	Critical Reading of Research Publications Plus course, USA, year(s) of implementation not specified	Program description: 6-week course comprising weekly 90-minute lessons delivered by a research nurse and doctoral student Workbook provided Curriculum: Research article introduction, design and sample, data collection, descriptive statistics, and inferential statistics Aim: To improve participants’ research self-efficacy	17 Nurses 1 hospital	Collaborative learning Experiential learning Mentoring Self-efficacy theory
Turkel, Ferket, Reidinger, & Beatty, 2008 [90]	Nursing Research Fellowship Program, USA, 2005–2006	Program description: 1-year long fellowship with 4 embedded educational workshops, each 8-hours in duration, with work to be completed outside of and between workshops Curriculum: Identifying a research problem, reviewing the literature, writing a research question, qualitative and quantitative research methods, replication studies, research proposal, research design, population sampling, data collection, ethics, statistics, and participant consent Aim: To advance excellence in professional nursing practice and research	7 Nurses 1 community hospital	Didactic learning Experiential learning Mentoring
Varnell, Haas, Duke, & Hudson, 2008 [62]	Accelerated EBP Educational Program, USA, 2006	Program description: 8-week program comprising 2-hour classes each week Content delivered by local university faculty members Curriculum: Introduction to EBP, asking clinical questions, basic research design, literature searches, critical appraisal, applying evidence in practice, and evaluation Aim: To increase nurses’ self-reported EBP beliefs and implementation	51 Nurses 5 acute care facilities	Didactic learning Experiential learning Transtheoretical model of change
Wells, Free, & Adams, 2007 [80]	Nursing Research Internship Program, USA, 2004–2005 (evaluation period)	Program description: 2-year program for selected nurses (identified by managers), comprising monthly workshops and self-directed learning in between. Mentoring/access to experienced nurse researchers was also inherent in the program Curriculum: Introduction to continuous quality improvement, systematic data collection, literature searching, critically analysing and synthesizing research papers/findings (in first year) Second year workshops were focused on implementing practice change, data management and research dissemination (abstract and manuscript preparation) Aim: To increase nurse interns’ research literacy, facilitate EBP and reduce barriers to EBP	17 Nurses 1 university hospital	Collaborative learning Didactic learning Experiential learning Mentoring
Wojtecki, Wade, & Pato, 2007 [48]	Teaching Practice-Generated Research Skills, USA, year(s) of implementation not specified	Program description: Ten 1-hour classes offered in weekly succession at the end of the workday. Facilitated by a biostatistician and a clinical nurse specialist Curriculum: Research terminology, basic statistics, developing a research question and a research project Aim: To teach clinicians how to conduct practice-generated research, with a view to enhancing evidence-based medicine knowledge and skills	14 Mixed 1 hospital	Didactic learning
**Program delivered between 2011 and 2020 (*n =*** **33)**				
Awaisu, Kheir, Alrowashdeh, et al., 2015 [63]	Pharmacy Practice Research Capacity Building Programme, Qatar, year(s) of implementation not specified	Program description: Intensive 26-hour training program delivered over four (weekend) days Training delivered via didactic lectures, case-based learning, group discussions, and self-directed learning. Course delivered by nine pharmacy research experts Curriculum: Ethics in human research, research design and methodology, critical appraisal of literature, data collection, biostatistics, and research dissemination Aim: To provide participations with knowledge and skills to plan and conduct a research project	24 Pharmacists 1 hospital and 1 cancer research centre	Collaborative learning Didactic learning Experiential learning
Berthelsen & Hølge-Hazelton, 2016 [49]	Research Education Intervention, Denmark, 2013–2014	Program description: Four teaching sessions delivered fortnightly (modified from 6 due to less than expected participation) Curriculum: Introduction to the program and general overview of nursing research; theoretical and methodological approaches Aim: To increase research usage among the nurse participants and aimed to develop the nurses’ theoretical and practical knowledge of research	32 Orthopaedic nurses 1 regional hospital	Didactic learning
Black, Balneaves, Garossino, Puyat, & Qian, 2015 [89]	Research Training for Point-of-Care Clinicians, Canada, 2011–2013	Program description: 18-month – 2-year long program whereby interested teams submitted a letter of intent which detailed research problem and team members Approved teams participated in 3 mentored training workshops Curriculum: Research methods, ethics, and literature review techniques Aim: Improve clinicians’ EBP and research knowledge, attitudes, and practices	153 Mixed 1 health organisation	Experiential learning Mentoring
Carey, Trout, & Qualls, 2019 [66]	Nurse Research Internship, USA, 2013–2015	Program description: Intensive, 9-month paid research internship for qualified nurses to bring their research question to a venue that supports to design, implement, and disseminate projects. Assistance provided by a nurse scientist Interns attend approximately 20 research classes with classroom quizzes cotaught by a nurse scientist and librarian Curriculum: Developing a clinically relevant research question, identifying key words, and conducting a literature search Aim: To build nurses’ capacity to frame research questions, search the evidence, and critically appraise the evidence	18 Nurses 1 hospital	Didactic learning Experiential learning Mentoring Quizzes
Chan, Glass, & Phang, 2020 [50]	Nursing Research and EBP Mentorship Program, Singapore, 2015	Program description: Mentorship program comprising classroom teaching, hands-on session, and one-on-one mentorship with an experienced researcher Mentees also conducted journal clubs, coached ward nurses on their projects, and were assigned a buddy to guide and co-lead together Curriculum: Classroom teaching included framing research questions, literature search, EBP principles, study designs, critical appraisal, biostatistics Mentoring focused on developing research, project management, evaluating project, data analysis, writing for publication Aim: To develop frontline nurses into EBP champions in their respective departments	9 (mentees) and 185 (ward colleagues) Nurses Acute care tertiary hospital	Collaborative learning Didactic learning Experiential learning Roger’s Diffusion of Innovation Theory Mentoring
Donley & Moon, 2021 [67]	Flexible Research Program for Social Workers, Australia, 2019	Program description: A 7-month research education program developed by a social work research lead, focused on research foundations Comprised of monthly email and brief oral presentations at team meetings (“Ten Minute Tips”) Second experiential phase involved the development of a research or quality assurance project with management and mentoring support Curriculum: Formulating a research question, literature review basics, ethics applications, methodology and analysis, formulating conclusions and presentation skills Aim: To increase social workers’ confidence to conduct research	30 Social workers (Allied health) 1 large inner-city public hospital	Didactic learning Experiential learning Mentoring
Duncanson, Webster, & Schmidt, 2018 [51]	Writing for Publication Bootcamp Australia, 2012–2015	Program description: Writing for publication bootcamp (WFP) for novice researchers was a structured additional (voluntary) component of a broader RCB program Six 1-hour sessions were held via teleconference weekly at 8 am and were facilitated by a program manager Program involved teaching, practical application, homework activities, peer interaction (and review) and facilitator support Curriculum: Teaching and practical experience in each stage of the WFP process Aim: Program aimed for 50% of novice researcher participants to submit a manuscript for publication. Other objectives were to increase participants’ knowledge, experience, and confidence in submitting a manuscript; deliver a program to rural participants and to make it cost-effective	50 Mixed Rural and regional public health services	Collaborative learning Didactic learning Experiential learning
Edward & Mills, 2013 [39]	Research outreach ward-based seminar (ROWS) program, Australia, 2011	Program description: ROWS program comprised part of a broader hospital-based research enhancement model ROWS program was delivered in an express (15 min) format in the ward setting for increased accessibility and attendance Managers attended or directed staff to attend to indicate their support Seminars were developed by nurse academics using and were aimed at nurses at all levels of education Curriculum: Seminar topics included locating research papers online, research in nursing and midwifery, critical analysis of research, ethical considerations, quantitative approaches and qualitative methods Aim: To increase nurses’ awareness of, access to, and use of research in the clinical context	197 Nurses and midwives Hospital (ward) setting	Didactic learning
Elkassem, Pallivalapila, Al Hail, et al., 2013 [43]	Pharmacy Practice Research Training Workshop, Qatar, 2011	Program description: Two consecutive day workshop delivered by pharmacy practice academics and researchers Curriculum: Research questions, critical appraisal of literature, developing research methods, data collection and analysis, disseminating findings Aim: To improve participants’ views and attitudes towards research	47 Pharmacists (primarily) 1 hospital	Collaborative learning Didactic learning
Famure, Batoy, Minkovich, Liyanage, & Kim, 2021 [44]	SPICE+B, Canada, 2013–2018	Program description: 10-week seminar series comprising didactic lectures and interactive review and discussion of research literature Lectures supplemented with online closed-access resources including lecture slides, audio recordings, and practice questions, and a participant discussion forum Curriculum: Clinical research methodology and design (e.g., bias, observational study design, clinical trials) Aim: To increase participants’ knowledge and ability to critically appraise medical research	750 Mixed Multicentre University Health Network	Collaborative learning Didactic learning Experiential learning
Friesen, Comino, Reath, et al., 2014 [81]	Primary and Community Health Research Unit (PCHRU) Researcher Mentoring Program Australia, 2011–2012	Program description: 12-month researcher mentoring program, including four research skill development workshops comprising didactic content and hands-on learning Six project teams were paired with a university-based research mentor to assist in completing a clinically relevant project Manager’s support was required as part of a competitive application process Curriculum: Workshop topics included developing a research question, data-collection tools, statistical analysis, disseminating research findings Aims: Broader program aimed to build research capacity and generate research evidence by and for primary and community health services Workshops aimed for novice researchers to present or publish the findings of a project	32 Mixed 1 local health district in a metropolitan area	Didactic learning Experiential learning Mentoring
Fry & Dombkins, 2017 [52]	Researcher Education Program, Australia, 2012–2015	Program description: Researcher Education Program was part of a broader multimodal program to build workforce capacity and leadership Education component comprised of six study days and ten 2-hour master classes. Research mentoring was a component of the broader RCB strategy Curriculum: Study days focused on developing a research idea, proposal, data collection, analysis, grant writing and publication Master classes provided practical information about data management, analysis, ethics applications, and use of common research software Aims: Broader program aimed to support nursing and midwifery research and leadership skill development to influence practice change, by addressing identified barriers to research Education program aimed to promote nurses’ capacity to understand, translate, utilise, and conduct research	> 2000 Nurses (primarily); study days and master classes also open to allied health, medical and administrative staff 1 metropolitan local health district	Didactic learning Experiential learning Knowledge to action framework Mentoring
Gardner, Smyth, Renison, Cann, & Vicary, 2012 [68]	Research Education Intervention, Australia, year(s) of implementation not specified	Program description: A 6-month multimodal education program comprising initial face-to-face workshops, further face-to-face support, videoconferences, informal email and phone support, and paper-based resources Multidisciplinary team of both nurses and librarians provided the workshops and ongoing support Participants were asked to prepare and submit a research proposal within the 6 months Curriculum: Not described Aim: To promote locally relevant clinical research activity and nurses’ attitudes to and orientation towards research in rural and remote settings	15 Nurses 2 healthcare sites in rural and remote settings	Didactic learning Reference to Roger’s Diffusion of Innovation theory Mentoring
Ghirotto, De Panfilis, & Di Leo, 2020 [82]	Qualitative Research Methodology and Methods (QRM) Training Program, Italy, 2015	Program description: Year-long multifaceted qualitative research methodology (QRM) training program, developed and delivered by 2 QRM experts Participation was voluntary, participants were required to have basic comprehension in research methodology Training program was 120 hours in total and comprised of lectures, classwork, group and individual work, simulations, and practical application where participants conducted a research study in groups. An e-learning platform was also available Curriculum: Knowledge and skills required to conduct qualitative research in all its steps (using grounded theory methodology) Aim: To enable health professionals to perform qualitative research within their work environment	14 Mixed 1 Clinical Cancer Centre	Collaborative learning Didactic learning Experiential learning
Harvey, Barker, & Tynan, 2020 [9]	Writing for Publication Program, Australia, 2018	Program description: Three 90-minute face-to-face writing workshops, delivered over an 8-month period by two researchers. Workshops commenced at 8 am to minimize the impact on workday Participants had not previously published as lead author Workshops comprised instruction on how to write for publication, strengths-based problem-solving, 30 minutes of writing, peer review and mentoring by experienced researchers Curriculum: Manuscript planning and preparation guidance, academic writing instruction, peer review Aim: To increase the capacity of allied health practitioners to write and submit manuscripts for publication in peer-reviewed journals	9 Allied health 1 regional public health service	Didactic learning Experiential learning Mentoring
Horstman & Theeke, 2012 [32]	Structured Professional Writing Retreat, USA, year(s) of implementation not specified	Program description: A 1-day (8 hours) intensive professional writing retreat, held off-site on a Saturday Retreat conducted in a conference-like setting with one round table per writing group. Post-retreat support with consultants to encourage continuation of work was also provided An external consultant was engaged to provide the content for the retreat. During the workshop writing groups developed purpose statement for an article, abstract, draft query letter, outline and finalized work plan. Curriculum: Practicalities of academic writing including, choosing a topic, focusing the paper, style, selecting a journal, writing, concept map, elements of good writing, ethical and legal issues, submission and review process, writing the work plan, writing a contract Aim: To improve nurses’ professional writing skills for publication and presentation with a view to increase nursing research publications	10 writing groups (of 4–8 participants) Nurses 1 hospital	Collaborative learning Didactic learning Experiential learning Mentoring
Johnson, Black, & Koh, 2016 [64]	Practice-Based Research Challenge, Canada, 2011–2015	Program description: 1-year program supported by volunteer research mentor (clinical specialists or academics) plus research skills workshops (1–4 hours duration) and statistics support Curriculum: Conducting a literature review, overview of research methods and research ethics Aim: To increase participants’ knowledge of research methods and enhance patient care through evidence-based practice	22 Dietitians (Allied health) Multisite healthcare organisation	Experiential learning Mentoring
Landeen, Kirkpatrick, & Doyle, 2017 [86]	Hope Research Community of Practice (HRCoP), Canada, 2015–2016	Program description: A year long program with monthly 3-hour seminars delivered by PhD-prepared nurses and mentoring support from biostatistician Curriculum: Topics covered included defining research question, choosing methodology, ethics application, research logistics, using a data collection/organisation tool, data analysis, planning for dissemination and translation Aim: To develop participants’ confidence and competence to complete research projects	7 Nurses 1 large multisite teaching hospital	Collaborative learning Didactic learning Experiential learning Mentoring
Lizarondo, Grimmer-Somers, Kumar, & Crockett, 2012 [70]	*i*CAHE Journal Club, Australia, year(s) of implementation not specified	Program description: Six 1-hour long journal club sessions using the *iCAHE* (International Centre for Allied Health Evidence) structured format, held monthly Groups nominated two facilitators who were required to attend a once-off training workshop. Facilitators were, in turn, instructed to train their members Curriculum: Included aspects of EBP such as formulating clinical questions, developing a search strategy, critical appraisal, evidence implementation and evaluation. Aim: To improve participants’ EBP knowledge, skills, and behaviour	93 Allied health 1 healthcare facility	Adult learning principles Collaborative learning Experiential learning
Mason, Lambton, & Fernandes, 2017 [92]	Clinical Nurse Research Fellows Program, USA, year(s) of implementation not specified	Program description: 1-year long program with monthly formal classes with a Professor Emerita and protected research time Letter of support from manager required as part of application Fellows present their project in the final class which is open to all healthcare providers and leaders Curriculum: Ethical conduct of research with children, protocol design, methodology, statistics/data analysis, institutional review board proposals, grant proposals, and manuscript preparation Aim: To provide clinical nurses with the skills to complete a research study	6 Nurses 1 paediatric hospital	Collaborative learning Experiential learning Mentoring
Mazzella-Ebstein, Barton-Burke & Fessele, 2020 [83]	Nursing Research Fellowship, USA, 2016–2019	Program description: 18-month research fellowship comprising 18 days of class time presented monthly (online modules and in-person) and mentoring with an experienced nursing researcher. Included protected research days over the first nine months and documented research idea and letter of support from manager/administrator Curriculum: Classes focused on supporting fellows to develop their study protocol for ethics submission Mentoring component focused on developing abstracts and manuscripts Aim: To engage nurses in the research process to facilitate new knowledge and innovations to improve patient care through symptom management	21 Nurses 1 Comprehensive Cancer Centre	Didactic learning Experiential learning Mentoring
McNab, Berry, & Skapetis, 2019 [53]	Research Education Lecture Series, Australia, year(s) of implementation not specified	Program description: Series of six 1-hour face-to-face lectures, delivered fortnightly over a 10-week period. Participation was voluntary Curriculum: Lectures included introduction, purpose/ definition of research, conducting research, ethics and governance and dissemination Aim: To promote understanding and development of research in hospital employees and to increase participants’ experience and intent to conduct to research	160 Mixed 1 tertiary referral hospital	Didactic learning
Mickan, Hilder, Wenke, & Thomas, 2019 [56]	Tailored EBP Education, Australia, year(s) of implementation not specified	Program description: 4-month intervention consisting of monthly 2-hour workshops, delivered by two academic researchers to small groups of participants (8 in each) Workshops consisted of short informal teaching, with practical group activities and discussion Curriculum: Steps of EBP, formulating answerable research questions, critically appraising research papers, applying evidence in practice Aim: To increase participants’ EBP self-efficacy, knowledge, and skills; integrate learnings about EBP into practice, and increase self-reported EBP behaviour	16 Allied health 1 hospital	Didactic learning Collaborative learning Experiential learning Self-efficacy theory
Mudderman, Nelson-Brantley, Wilson-Sands, Brahn, & Graves, 2020 [75]	EBP Education and Mentoring Program, USA, year(s) of implementation not specified	Program description: 5-month program comprising 8 sessions including 7 lectures, independent work time, and a final session to disseminate their findings The lectures were between 30 and 150 minutes in duration, held at midday to accommodate different shifts and were recorded Curriculum: Introduction to EBP, appraise and synthesise literature, design and pilot practice change, integrate and sustain practice change Aim: To improve the knowledge, practice, and attitudes toward EBP among staff nurses and clinicians in a rural CAH	10 Mixed 1 rural critical access hospital	Collaborative learning Didactic learning Experiential learning Mentoring
Munro, Tacchi, & Trembath, 2016 [45]	Course on Research Skills (Pilot), England, year(s) of implementation not specified	Program description: 11 research education sessions as standalone units or a complete course Curriculum: Reflect the concept of compassion in practice and includes the history of research, research methodology, clinical research nursing, protocol review and feasibility, audits, patient centredness, patient information, informed consent, participant recruitment, study management, data entry, documentation Aim: To develop participants’ expertise in adhering to complex clinical trial protocols; ensure robust quality systems and documentation	77 Nurses 4 (pilot) sites	Didactic learning
Saunders, Vehviläinen-Julkunen, & Stevens, 2016 [65]	EBP and Research Utilization Education, Finland, 2014–2015	Program description: 4-hour live session consisting of didactic learning delivered by advanced practice nurses with EBP expertise Access to web-based educational materials on an interactive learning platform and mentor support for 8 weeks Curriculum: EBP concepts (locating, critically appraising, and summarizing the evidence) and research utilization (integrating evidence into decision-making, measuring outcomes, and implementing EBP change) for the intervention and control groups Aim: To enhance nurse participants’ readiness to for EBP	77 Nurses 1 university hospital	ARCC (based on cognitive-behavioural theory and control theory) Didactic learning Experiential learning Mentoring
Schmidt, Webster, & Duncanson, 2019 [85]	Rural Research Capacity Building Program, Australia, 2006–2013	Program description: Experiential research education program delivered over 2-year period comprising 10 full-day in-person education sessions, weekly teleconferencing, and mentoring Competitive application process undertaken which required participants to submit a research proposal endorsed by their organisation Clinical backfill (60 days over 2 years) Curriculum: Understanding research, writing a research protocol, research methods, and research report-writing Aim: To increase rural health research capacity	167 Mixed Multiple rural and regional health services	Didactic learning Experiential learning Mentoring
Schmidt & Kirby, 2016 [93]	Centre for Research Excellence Rural Research Capacity Building Program, Australia, 2014 (program linked to that described by Schmidt et al. [85]	Program description: Modular short course delivered followed by small group meetings Mentoring and support for 2-years Curriculum: Quantitative research (introduction to statistics, measures of frequency and association, questionnaire design), qualitative research (interviews, focus groups, coding, and analysis) critically reviewing the literature, and project development Aims: To build individual and organisational health services research capacity and to build meaningful relationships between university departments of rural health and healthcare providers Trainees were expected to develop and present a formal research report and submit a paper for per-reviewed journal publication	7 Mixed 3 sites	Collaborative learning Didactic learning Mentoring
Tilson & Mickan, 2014 [72, 102]^a^	PEAK (Physical therapist driven Education for Actionable Knowledge translation) Program, USA, year(s) of implementation not specified	Program description: Multifaceted 6-month program including a two-day training workshop delivered by a clinician- researcher and librarian, who also provided ongoing support to participants for 4 months Curriculum: Literature searching, using technology for EBP, critical appraisal, evidence synthesis, adapting evidence to local context, and selecting topic for knowledge translation Aim: To improve participants’ EBP attitudes, self-efficacy, knowledge, skills, and behaviour	18 Physiotherapists (Allied health) 3 patient care centres	Collaborative learning Didactic learning Experiential learning Mentoring Promoting Action on Research Implementation in Health Services (PARiHS) Framework Social cognitive (self-efficacy) theory
Tsujimoto, Kataoka, Sato, et al., 2021 [76]	Systematic Review Workshop, Japan, 2015–2017	Program description: 6-month program comprising a combination of seven short lectures, homework, discussions and feedback and support from facilitators Curriculum: Developing systematic review questions ad search strategies, using bibliographic software, establishing inclusion and exclusion criteria, assess for risk of bias, perform meta-analyses, narrative synthesis, quality appraisal, review registration, and dissemination Aim: To provide healthcare staff with skills to create systematic review protocols based on their own clinical questions at teaching hospitals	233 Mixed 9 hospitals	Collaborative learning Didactic learning Experiential learning
Wenke, Thomas, Hughes, & Mickan, 2018 [69]	TREAT (Tailoring Research Evidence and Theory) Journal Clubs, Australia, year(s) of implementation not specified	Program description: Five monthly journal clubs using a structured format: “TREAT”, which incorporated eleven ‘key components’ of successful journal clubs Facilitated by academic allied health researchers experienced in teaching and using EBP who were also available for mentoring support between sessions Curriculum: Goal setting to identify relevant topics, use if PICO approach to clarify clinical questions, group critical appraisal using structured “Critical Appraisal Skills Programme”, tools, and engaging librarian support Aim: To improve allied health professionals’ EBP skills	61 Allied health professionals 1 large health service	Adult learning principles *not specified* Collaborative learning Didactic learning Experiential learning Mentoring
Wilson, Ice, Nakashima, et al., 2015 [54]	Hybrid Model Journal Club, USA, year(s) of implementation not specified	Program description: Multidisciplinary bi-weekly journal clubs conducted in-person and online using a secure social media site, over an 8-week period (four sessions in total) Sessions facilitated by a PhD-prepared nurse and followed four steps of EBP: Ask, access, appraise, apply Participants could earn contact hours/points towards a clinical ladder program Curriculum: The journal club was designed with the general topic of “Improving Pain Management”; curriculum focused on clinical problem solving Aim: To increase EBP skills, self-efficacy, research use, behaviours, ability, desire and decrease reported barriers	36 Nurses (primarily) 1 large urban hospital	Collaborative learning Didactic learning Experiential learning Self-efficacy theory
Withington, Alcorn, Maybery, & Goodyear, 2020 [84]	Training and Mentoring Program, Australia, year(s) of implementation not specified	Program description: 3-year multimodal program comprising 2-day face-to-face training session followed by monthly group and individual mentoring, delivered by a university Additional supports provided by health service (steering committee review, access to consultation and online educational material) Placements in program limited and competitive application process undertaken Participants required to complete a service evaluation or research project Curriculum: Designed to walk participants through steps of a research project including design, ethics, implementation, analysis and write up Aim: To build clinicians’ research capacity with senior level support within the organisation	21 Social workers (Allied health) 1 large paediatric health service	Collaborative learning Mentoring

^a^PEAK program is described in [72] and the evaluation is reported in linked paper [102]

#### How research education programs were formatted and delivered

Research education programs were delivered in several different formats and over different types of durations. Some were delivered as standalone single study days, workshops or sessions [29–34], and others as a series of several short sessions or workshops [35–45]. The majority of papers described integrated research education courses of either a short duration, (i.e., one to 4 months) [46–65], medium duration (i.e., five to 11 months) [9, 66–76], or longer-duration (i.e., 1 year or longer) [77–94].

Programs almost always included a didactic element (e.g., lectures, seminars), delivered by an experienced academic or clinician-researcher (researcher with a primary healthcare qualification; [95]) or an individual with content expertise (e.g., biostatistician [48], librarian [33, 57, 66], ethics committee member [57] or data manager [42]). Most of the programs were multifaceted and included a mix of didactic teaching as well as either group discussion, online teaching (e.g., teleconferences or modules), or the practical application of theoretical principles between education sessions. Several were described as single mode research education programs (e.g., seminars, lectures, or online modules only) [29–31, 33, 37–39, 46, 48, 49, 53–55, 87]. Timing was described as an important consideration in several papers, with an emphasis on minimising impact on participants’ working day or clinical duties. For example, by holding sessions early (8 am) prior to the working day [9, 51] or on weekends [32, 63, 71].

#### Features and content of research education programs

The curricula or research education content described in the papers reflected the aims of the programs. Program aims were broadly categorised according to the level of intended participants’ research engagement: research use or consumption (*n =* 28) and research activity (*n =* 31) [96]. Where the program content focused on searching, retrieving, and appraising research literature, and considering in the context of clinical practice (i.e., evidence-based practice), this was considered engagement at the research user or consumer level. Slightly more programs were concerned with developing research skills to engage in and conduct research activity. These programs included content related to research methods, data collection and analysis techniques, protocol development and ethics application [31, 35, 37, 39, 42, 43, 48, 49, 52, 53, 57, 59, 63, 64, 67, 68, 73, 77–85, 90–92]. Seven programs were orientated toward developing participants’ skills for research dissemination, typically writing for publication [9, 32, 33, 47, 51, 74] or preparing research posters and seminars [88]. It was assumed that the participants in the programs concerned with writing for publication had already undertaken a research activity and needed further education and support to formally disseminate their findings. Two programs were specifically focused on developing participants’ skills to complete a systematic review [46, 76]. Three programs included content directly related to implementing research in practice [60, 80, 86].

Fourteen programs required that participants had overt support from their manager to participate (e.g., written approval or direct selection of participants) [46, 51, 58, 62, 75, 79–81, 83, 85, 91–94]. Two papers described participants’ departments being actively supportive of their participation in the research education program [59, 86]. One paper referred to managers’ positive role modelling by engaging in the research education program [39] and another described the criteria used to determine the suitability of participants based on their context (i.e., supportive managers who were interested in research and willing to release participating staff for half day each week) [88]. Five papers described manager or leadership support as being a key enabler to participants engaging in the education program [56, 60, 75, 89, 91] and four papers referred explicitly to the lack of organisational, managerial, or collegial support as key limitations to, or a negative influence on participants’ learning experience [49, 77, 84, 88].

Nine papers described the integration of opportunities to acknowledge the achievements of program participants. Opportunities were described as formal events held at the conclusion of the program to celebrate the participants’ completion [58, 66, 80, 83], recognition via staff communications or at an organisation-wide event [37], opening participants’ project presentations to a wider healthcare organisation audience [92], or by managers providing opportunities for participating staff to present their work to colleagues [81, 82]. One program included the acknowledgment of contact hours for nurse participants to attain continuing professional development points for their professional registration [54] and another referred to participants’ “recognition and exposure” within and beyond their organisation, as a participant-reported benefit (46, e–145).

#### Theories and pedagogical principles

Understanding how people learn effectively is fundamental to the design of any educational program. Thus, the second aim of this review was to determine what pedagogies (teaching methods) were employed for adult learners undertaking research education and training. Few of the studies (*n =* 13) included in this review explicitly stated which pedagogical strategies informed the design and delivery of the education programs. However, where possible we extracted pedagogical strategies that appear to be present (see Table 2).

Education programs generally included a mix of active and passive learning strategies. Active learning can be defined as an activity which engages students as participants in the learning process whereas with passive learning, students receive information from the instructor but have little active involvement [97]. Passive forms of learning or didactic approaches that were employed included seminars, lectures, reading, and exams. Five programs were described with respect to the didactic learning component only, with no reference or implication of any underlying pedagogy or learning theory [39, 45, 48, 49, 53].

Commonly, education programs included some form of experiential learning. Experiential learning, or “learning by doing” is a type of active learning whereby students apply knowledge to real-world situations and then reflect on the process and experience [98]. Examples of experiential learning described in the education programs include simulations, role-play, preparation of research protocols, grant proposals, manuscripts, and appraisal of research. Lack of experiential learning, or “practical experience”, was described as a limitation in one paper [38]. Quizzes were utilised in two programs [42, 66] to reinforce participants’ learning.

Social cognitive theories of learning, such as self-efficacy theory [99], were explicitly mentioned in seven studies [31, 47, 54, 56, 61, 71, 72]. Self-efficacy theory posits that a person’s belief in their capabilities provide the foundation for performance and accomplishment. If a person has low self-efficacy (little belief in their capabilities) and fear related to the task at hand, they will likely avoid that task for fear of failure. Education programs using a self-efficacy framework focused on increasing participants self-efficacy through coaching, support, social modelling, and mastery experiences. Five studies referred to Roger’s Diffusion of Innovation theory [37, 50, 60, 68, 71], which posits that identifying and working with highly motivated individuals is an efficient way to promote the adoption of new behaviours and practices more widely [8].

Two studies were informed by the Advancing Research and Clinical practice through close Collaboration (ARCC) Model which is based on cognitive-behavioural theory and control theory, and therefore designed to address barriers to desired behaviours and practice [65, 100]. Other programs described drew on the transtheoretical model of organisational change [62], Donald Ely’s conditions for change [37], the knowledge to action framework [52] and the Promoting Action on Research Implementation in Health Services (PARiHS) Framework [72].

Mentoring was a feature of more than half of the programs (*n =* 37). This is where novice researchers were paired with an experienced researcher, typically to support their application and practice of the knowledge gleaned through their education or training [101]. In three papers describing programs that did not include mentoring, this was identified as a critical element for future research education programs [37, 78, 92]. Several evaluations of programs that included mentoring illustrated that it was required throughout the life of the program and beyond [9, 32, 67, 68, 73, 81, 84]. Harding et al. [46] found that mentors as well as mentees, benefited from the research education program, in terms of their own learning and motivation.

Social theories of learning, or collaborative learning approaches, were also frequently utilised (*n =* 40). Collaborative learning approaches are based on the notion that learning is a social activity at its core, shaped by context and community. Such approaches promote socialisation and require learners to collaborate as a group to solve problems, complete tasks, or understand new concepts. Collaborative approaches utilised included journal clubs [38, 50, 54, 69, 70, 87], writing groups [32, 51], classroom discussions [33, 36, 72, 76, 80, 94], interactive group workshops or activities [29, 31, 46, 47, 56, 75, 82, 84, 86, 93], and development of team research projects [78, 79]. These approaches were often reported to enhance cultural support with participants networking, sharing resources, and celebrating successes together. One program employed a self-guided learning approach through the use of computer-based learning modules [55].

### Approaches to program evaluation

Less than half of the included papers accurately and comprehensively described the methodology and methods used to evaluate the research education program [9, 30, 38, 46, 54–56, 60–63, 65, 69–71, 75, 77, 79, 82, 84–86, 89, 100, 102]. The remaining papers either referred to the data collection techniques used without describing the overarching approach or methodology. Therefore, in Table 3 rather than referring to the approach to program evaluation as quantitative, qualitative or mixed methods, reference is made to the data collection techniques (e.g., surveys, interviews, facilitator reflections, audit of research outputs).

**Table 3:** Research education program evaluation and outcomes reported

Program	Evaluation data collection method and sample size	Primary outcome	Secondary and other outcome/s	Key findings
**Program delivered before 1991 (*n =*** **1)**				
Research Nurse Internship [94]	Author’s observations *n =* not stated	Observed outcomes	Participant feedback (informal)	14 participants have pursued further formal nursing education, 22 presented at a national or local research or clinical practice conference Participants’ projects have led to impactful changes to clinical practice Participants’ feedback indicated the internship was useful in strengthening the link between research and practice, led to increased job satisfaction and a mechanism to develop clinical and research networks
**Program delivered between 1991 and 2000 (*n =*** **9)**				
Basic Research Methodology for Nurses [77]	Interviews combined close-ended and open-ended questions generating quantitative and qualitative data. *n =* 37 intervention *n =* 42 control	Self-reported research activity	Self-reported interest or commitment to research	Participants planned to engage in research Some completed research and published findings Research knowledge is important but not sufficient to realise more nurse-led research activity
Critical appraisal of research workshop [29]	Participant evaluation survey *n =* 23	Satisfaction with program	Program evaluation	Workshop participants increased understanding of how research improves patient care Improved attitudes towards EBP Participants valued small group discussion
Research Training Workshops [35]	Informal quantitative data collection *n =* N/A	Summary of research outputs/ outcomes (grant funding secured, journal publications, conference papers, external grant funding)	N/A	Workshop participation led to external research grant funding success and nurse-led research publication in peer-reviewed journals
Critical Research Reading Skills Study Day [30]	Pre- and post- intervention surveys *n =* 19	Critical appraisal of research skills (measured objectively using inter-rater comparison)	Self-reported frequency of use of published research papers (pre- and post-intervention)	Participants’ research critical appraisal skills increased Participants reported reading research more frequently and with greater confidence than before the study day Brief study days contribute to increasing nurses’ use of research in practice
Raising Research Awareness Among Midwives and Nurses [36]	Pre- and post-intervention survey *n =* 259 (intervention) *n =* 131 (control)	Awareness of research	Attitudes toward research and toward nurses who do research Barriers to reading or doing research Knowledge and use of research resources	Intervention led to increased self-reported use of research Staffing levels are a significant barrier to nurses doing research and that an enabling environment is critical It is integral to train and retain research-capable nurses
Dissemination and Utilization of Research Findings in Clinical Nursing Practice [88]	Focus groups *n =* 10 (2 groups of 5, repeated either 9 or 10 times, throughout program)	Participants’ experiences of disseminating and implementing research in their setting		Organisational factors including workload, resources, competing priorities, other changes and level of manager interest and support, influenced participants’ capacity to disseminate and implement research into practice Becoming a change agent was challenging and accompanied by feelings of guilt and that implementing EBP is not seen as real work by colleague Some wards were supportive of innovation Manager support, leadership, and a learning culture are critical to participants’ ability to apply their new research knowledge and skills in practice
Research Utilization Workshops [41]	Pre-workshop interview Post-workshop focus groups Post-workshop survey *n =* not stated (pre-workshop interview) *n =* not stated (post-workshop focus groups) i173 (survey)	Satisfaction with program (workshop content, presentation, value, meeting objectives)	Perceptions of research Self-reported confidence and skill development	Most participants rated the workshop highly, would recommend to a colleague, and considered the objectives were met Participants developed positive attitudes towards research, felt motived, and perceived that their research skills were strengthened following the workshop
Research Workshop [31]	Pre- and post-intervention survey i31	Self-efficacy (perceived ability to participate in or initiate research)	Subsequent development of nurse-led research projects	Participants reported enhanced research-related self-efficacy Some went on to engage in research activities Participating nurses may influence their peers to engage in research
Research Utilization Course [34]	Surveys post-course Interviews 6- and 12-months post course *n =* not stated (surveys) *n =* 21 (interviews)	Satisfaction with program	Self-reported outcomes of course Barriers and facilitators of project completion	Participants were satisfied with the course and valued the small class size and mentoring aspects Three participants completed projects as proposed, five were in progress, seven began a new course, nine engaged in EBP, five published papers, and two presented their projects at conferences Barriers to project completion included a lack of administerial support within the clinical environment, competing priorities and difficulties with implementation and sustaining practice change Facilitators to project completion included a supportive institutional environment, peer and multidisciplinary support, and autonomy
**Program delivered between 2001 and 2010 (*n =*** **25)**				
Practice Nurse Clinical Research Workshops [42]	Post workshop survey i89	Satisfaction with program	Self-reported confidence and competence to engage in research	Most participants considered the workshops to be ‘excellent’ or ‘good’; some reported feeling more confident and competent to engage in research Program provided pilot data for future research training and education programs
Research and Evaluation Capacity-Building Program in the Community Health Sector [37]	Cooperative action research drawing on data collected via informal conversations with staff, journal entries, interviews, and recordings of meetings *n =* not stated	Self-reported confidence and knowledge of how to undertake research and evaluation projects	Organisational capacity/ infrastructure (e.g., intranet page devoted to research and evaluation)	Training alone was insufficient to develop sufficient confidence and competence to undertake research independently Coaching, and mentoring is an important component in research capacity-building programs A framework aided the implementation the service wide RCB program
Research courses and journal clubs [38]	Survey (open and closed questions) at 3 time points (pre-, immediately post- and then one year after program) i81 control i89 intervention	Research knowledge (measured objectively)	Self-reported knowledge, skills, and attitudes Research-related activity	Intervention led to improved nursing research capability, knowledge, and skills Control groups nurses’ research skills and knowledge remained unchanged however, their attitudes towards research were significantly better after the intervention Intervention had an extended effect on all nurses, highlighting the influence contextual factors have on individuals’ research capability
Intensive Research and Management Capacity-building Program for Hospital Health Care Professionals [78]	Case study draws attendance data, characteristics of attendees, projects submitted, attendance and drop-out rates, satisfaction surveys inot stated	Submission / completion of research projects (i.e., final certification)	Satisfaction with course (surveys)	Program promoted active and enduring participation and influenced behaviour change Almost half of the research teams submitted a completed research paper Participants satisfied with course content
Publishing Short Course [33]	Survey i 32	Self-reported outcomes (perceptions of publishing, intention to use knowledge) and satisfaction with course		Average 4/5 for each domain; authors surmised that the course had little impact on participants’ perceptions of publishing Interaction/ discussion throughout the course indicated engagement with new knowledge of publishing
Leadership Journal Club [87]	Survey i20	Satisfaction with JC and self-reported outcomes (changes in research appraisal skills)	Engagement /attendance Tangible outcomes	Participants self-reported increased knowledge, satisfaction with setting (hospital) and competence of leader Areas for improvement were enhancing the environment for leadership decision making (2 goals of the journal club) and improved teaching methods Good engagement and attendance (approx. 20 at each JC) Three tangible outcomes: evidence-based fact sheet/recommendations for shift staff; 1 manuscript; and a statistical review of performance indicators presented by participants
12-Week Allied Health Research Training Scheme [46]	Pre- and post- survey (Research Spider tool) and interviews with participants and mentors i12 (6 mentors, 6 mentees interviewed) i7 mentees (survey)	Qualitative analysis of experiences and perceived benefits of the program (mentors and mentees)	Research confidence, experience, and interest (pre-and post-program)	Numerous participant-reported benefits of program including exposure to and recognition by colleagues within and beyond their organisation; networking with other clinicians from different disciplines, and influencing clinical practice through their research Mentors also benefited from the experience Program was too intensive for some participants; some were less supported by their manager/organisation Research confidence increased after the training One systematic review was accepted for publication, two were under peer review and another had an abstract published
Computer-Based Learning EBP Program [55]	Pre- and post-surveys (Evidence-Based Nursing Questionnaire) i744 (baseline) i314 (intervention/ post-training)	EBP knowledge, attitudes, and skills	Organisational readiness (for EBP)	Program led to increased self-reported EBP knowledge, attitude, and skill and their perceptions of organisational readiness for EBP Computer-based program negated the need for travel and provided for flexibility in meeting learners’ needs
Designated Research Team [79]	Non-randomised, matched-pair trial using the validated research capacity and culture (RCC) tool/survey pre- and post-intervention i37 (4 teams) intervention *n =* 32 (4 teams) control	Individual, team and organisational research capacity and culture domains	N/A	Program led to improved individual research skills and to a lesser extent, improved team, and organisational capacity to support research The more cogent impact on individual RCB attributed to the focus of the intervention on skill development and application to individual projects, as opposed to broader policy and practice change
Masterclass on Scientific Research Training for Public Health Professionals [91]	Surveys Focus groups Engagement and retention i16 (surveys) *n =* 16 (focus groups)	Satisfaction with masterclass content, organisation, and facilities	Participant experiences during the masterclass Self-reported skill development	Fourteen participants fulfilled the requirements for a masterclass certificate Fourteen draft manuscripts were underway, with more than 20 delivering oral presentations Participants had generally positive experiences of the masterclass and felt equipped and confident to conduct research Permission to attend the masterclass and social support from managers and colleagues were key facilitators to engaging in the course
Critical Appraisal Module [40]	Survey Author/facilitator reflections *n =* not stated	Satisfaction with program	Self-reported critical appraisal knowledge Self-reported perception of future impact on practice	Most participants rated the session 5 of 5, reported increased knowledge following workshops and perceived the workshops would impact clinical practice Timing and location of training supports health professional attendance Workshops deemed effective when participants are from mixed disciplines and engaged in a clinical scenario
QMC Nursing Research Fellowship [73]	Proposal submission Program evaluation survey *n =* not stated	Number of participants that submitted fellowship grant proposals	Satisfaction with program (quantitative measures and qualitative feedback)	Six fellows submitted a grant proposal, five were funded Fellows were satisfied that the program objectives were met and were satisfied with education providers/presenters Fellows required more individualized mentoring than was offered
Advancing Research and Clinical practice through close Collaboration (ARCC) Model [100]	Pre- and post- EBP Beliefs Scale, EBP Implementation scale, Group Cohesion Scale, Index of work Satisfaction (surveys) Nurse productivity audit Nurse attrition audit *n =* 22 intervention *n =* 24 control	EBP beliefs and practices	Group cohesion Job satisfaction Productivity Nurse staff attrition	The ARCC intervention group achieved significant improvements in their EBP beliefs and practices There were no significant differences between the ARCC and the control group regarding group cohesion, job satisfaction or productivity Less was attrition observed in the sites where the ARCC participants worked Mentorship is a key strategy for enhancing nurses’ EBP beliefs and implementation
Radiography Research Course [57]	Course evaluation form Author/facilitator reflections *n =* not stated	Satisfaction with program	N/A	Participants saw value in having the opportunity for independent study during the course but noted the course content was rushed at times Participants demonstrated commitment to research and clinical governance Authors reflected that the course resulted in strengthened relationships between involved organisations and increased awareness of relevant research for practice
Evidence-Based Practice Workshop [71]	Pre- and post-workshop adapted Fresno test and a bespoke survey Activity diaries *n =* 114 (baseline) *n =* 106 (post-training) *n =* 51 (8-months post)	EBP knowledge (objective)	Attitudes toward EBP and behaviour change	Program led to increased EBP knowledge and self-reported confidence in engaging in EBP Critical appraisal remained a challenge for many participants and research utilisation (behaviour change) was low at baseline and did not change after the program
Clinical Research Fellowship Programme [58]	Survey *n =* 8	Self-reported frequency of dissemination of project findings	Self-reported use of critical appraisal skills in practice Self-reported personal and professional program outcomes	Most projects were presented at national or international conferences and/or written into manuscripts for publication Most projects impacted clinician practice, except one where ward support was low Participants increased confidence to engage in a multidisciplinary research team. Some participants adopted critical appraisal in practice, took on research roles and completed research degrees following the program Participants felt safe, supported, and encouraged by the program cohort
Practice-Oriented Research Training (PORT) Program [59]	Survey *n =* 11	Self-reported research skill development	Qualitative feedback Grant submissions	Participants reported improvements in research skill Mentor support, grant/proposal writing, research fundamentals and sharing proposals were considered of most value Nine participants submitted grant and research proposals, all of whom had pre-existing research ideas
Speech and Language Therapy Research Training Program [60]	Pre-test post-test cluster RCT using a process-based audit tool to examine case notes Interviews to determine cost data types Strategy A: *n =* 325 patients (pre- training audit) *n =* 274 (post- training audit) Strategy B: *n =* 339 (pre-training audit) *n =* 304 (post-training audit) *n =* not stated interviews	Pre- and post-intervention audit of adherence to clinical guidelines	Resource requirement of the two strategies	Departments that received management training and critical appraisal training engaged more with research information although these practices did not impact changes in clinical practice six months following the intervention No relationship was observed between strategy cost and clinical outcome Management support for guidelines adherence and other organisational features may have influenced the findings
Writing for Publication Programme [74]	Writing outcomes Evaluation survey Focus groups Attendance records *n =* 37 (survey) *n =* 9 (focus groups)	Submissions to peer-reviewed journals	Satisfaction with program (quantitative measures and qualitative feedback) Experience of program Number of participants that attended four or more sessions	Four participants met the program objective: to publish a peer-reviewed paper and more than half were actively writing papers Participants valued to relaxed learning environment, peer and professional support Approximately half of the participants attended four or more sessions
Bedside to Byline [47]	Pre- and post- intervention surveys *n =* 11 (pre-intervention) *n =* 8 (post-intervention)	Writing self-efficacy	Manuscripts developed/ published Satisfaction with program	Program addressed barriers to nurses achieving scholarly publications Improved writing self-efficacy Participants preferred shorter workshops (i.e., 4 rather than 8 hours) and valued the peer learning environment Successful scholarly publication for some participants
Critical Reading of Research Publications Plus course [61]	Pre- and post-program Nursing Research Self-Efficacy scale (surveys) *n =* 17	Research self-efficacy	Satisfaction with program (qualitative feedback)	Program led to a significant increase in research self-efficacy in quantitative methods, using theory, and evidence Course enabled attainment of new knowledge and increased research confidence in positive environment A research role model promoted a sense of safety for participants to engage with research
Nursing Research Fellowship Program [90]	Pre- and post-intervention surveys Qualitative feedback Observed outcomes *n =* 7	Self-reported research knowledge and skills	Self-reported experiences of the fellowship program	Participants reported improved research knowledge and skills Fellowship projects led to numerous research outputs including conference presentations and publications The program led to observed evidence-based changes to practice
Accelerated EBP Educational Program [62]	Pre- and post-intervention surveys using the EBP Beliefs (EBPB) and EBP Implementation (EBPI) Scales *n =* 49	Self-reported beliefs about EBP	Self-reported implementation of EBP	Nurses that were and were not familiar with EBP prior to the program improved their awareness of EBP Administrative support was a positive influence on nurses’ willingness to engage in EBP Breaking learning opportunities into manageable components was considered beneficial
Nursing Research Internship Program [80]	Interviews (method) and then findings were quantified *n =* 10	Self-reported engagement in research or other research-related activities post-internship	Changes in attitudes toward research and practice change	Internship increased literature search activity, comfort in critically appraising research evidence, and in the application of research in practice Decreases in previously identified barriers to EBP Manager support was integral to the success of the program
Teaching Practice-Generated Research Skills [48]	Pre- and post- survey *n =* not stated	Attendance rate/ engagement	Research knowledge objectively evaluated (study design, statistics, and epidemiology) Research activity Research outputs (conference presentations)	Participants learned and applied new research knowledge to their own research activities Research knowledge increased post-training Engagement in the program was evident by consistent attendance and completion of tasks Local, clinician-led research activities were progressed because of the research skills classes
**Program delivered between 2011 and 2020 (*n =*** **33)**				
Pharmacy Practice Research Capacity Building Programme [63]	Pre- and post-program surveys *n =* 24	Objective measures of research skills	Self-reported research competency and confidence in research planning and conduct Training preferences	Mean overall increase in participants’ objective research skills Self-reported competence and confidence to plan and conduct research improved significantly Lack of time was reported as the main barrier to research activity
Research Education Intervention [49]	Surveys Focus groups *n =* 32	Self-reported relevance of education program to practice	Barriers (individual and contextual) to participation in the education program	Some participants developed new perspectives on nursing research in practice, others were unable to see the relevance of research to nursing practice Barriers such as personal factors and a lack of manager and collegial support, inhibited research engagement
Research Training for Point-of-Care Clinicians [89]	Pre- and post- surveys measured at three time points (baseline, 3-months post training, and completion of project) and focus groups/ interviews six months-post training *n =* 136	Self-reported research knowledge, willingness, and ability	Benefits, impacts of, and challenges associated with the education program	Research knowledge improved significantly; research ability improved at the completion of the project, but no significant improvement in willingness to engage in research was observed across the three survey timepoints Program provided an important opportunity for clinicians to learn about research and promoted excitement about research and clinical work Training was also perceived to benefit the organisation by showcasing research activity, promoting new collaborations, and increasing engagement in EBP Mentors were considered invaluable in helping participants navigate challenges associated with research and EBP
Nurse Research Internship [66]	Participant scores (pre- and immediately post-internship) and evaluation survey post-internship *n =* 14	Self-reported outcomes	Satisfaction with internship Pre- and post- internship quiz grade	Interns reported improved research skills Most had conducted literature searches, used library resources, analysed data, and participated in a journal club after the internship With the input and availability of the librarian, research internships increased nurses’ library-related research skills
Nursing Research and EBP Mentorship Program [50]	Pre- and 3-month post- mentorship program EBPQ survey *n =* 197 (pre) *n =* 194 (post)	Knowledge, attitudes, skills in EBP	N/A	Program mentees reported significant improvements in their EBP knowledge, attitude, and practice three months after the program Mentees’ colleagues also reported increased EBP knowledge and practice Mentees became EBP advocates, and this diffusion of knowledge led to improved organisational EBP culture
Flexible Research Program for Social Workers [67]	Pre- and post- program surveys *n =* 17 (pre) *n =* 12 (post)	Research confidence	Challenges while doing research Enablers of research activity Importance of being involved in research (thematic analysis)	Twelve individual or group projects were developed Participants’ self-reported confidence levels increased in all areas of research Challenges to research were limited time/competing priorities, limited skills, experience, and access to resources Enablers were access to the research lead, mentoring and active involvement in research Flexible approach to training delivery enhanced participant engagement
Writing for Publication Bootcamp (teleconference delivery) [51]	Writing outcomes audit Surveys *n =* 62 (control) *n =* 50 (intervention) *n =* 29 survey participants	Publication rates	WFP efficacy (knowledge, experience, and confidence) WFP bootcamp evaluation surveys Cost evaluation	Program led to increased publication rates for novice researchers Led to increased knowledge, experience, and confidence in writing for publication Participants valued the opportunity to share and receive critical feedback with and from peers and the facilitator High participant retention rate and satisfaction Cost of program per publication was $230
Research Outreach Ward-based Seminar (ROWS) program [39]	Survey *n =* 78	Satisfaction with program	Self-reported barriers and enablers to engaging in research activity Self-reported impact of program on clinical practice	Brief nature of the program helped participants overcome time as a barrier to engage with research and enhanced access to research training Participants recognised the importance of research in nursing practice Knowledge gap remains as to how to integrate EBP activities into clinical areas
Pharmacy Practice Research Training Workshop, Qatar [43]	Pre- and post-program survey i47 (pre) i37 (post)	Attainment of learning outcomes	Experiences of program Confidence to undertake research	The course was oversubscribed and well-attended Most participants agreed that all learning outcomes were achieved and felt confident to engage in research activity
SPICE+B [44]	Pre- and post- program surveys *n =* 730 (pre) i420 (post) i163 (impact, 1–5 years post-program)	Satisfaction with program content and delivery	Effect of program on participant development, practice, and future research engagement	Participants were satisfied with course content and delivery, particularly the hybrid in-person classes and online resources The short and longer-term evaluations were similar however in the longer term, many participants reported gaining the knowledge to pursue research opportunities Suggested improvements to the program include content on statistical software and greater emphasis on practice sessions
Primary and Community Health Research Unit (PCHRU) Researcher Mentoring Program [81]	Case study combining research outputs, participant feedback Focus groups *n =* 32 (6 project teams)	Research outputs (abstracts accepted, presentations, informal dissemination, and peer-reviewed publication)	Research activity (data collection and analysis) Participant-perceived research facilitators and barriers	Participating teams attained ethics approval, completed data collection, and commenced data analysis Enablers to research progress were supportive managers, networking, and mentor support Barriers to research progress were poor access to research infrastructure, lack of access to validated research tools, insufficient time, and difficulty navigating research ethics and governance systems
Researcher Education Program and Mentor Program [52]	Case study drew on data gathered via 1) study day and master class participant surveys and 2) pre- and post-program surveys of participants and mentors Authors reflect on some of the impacts of the program *n=* > 500 program participants	Program evaluation/ satisfaction with program	Self-reported research knowledge, confidence, and skills	Program increased participants’ confidence to pursue research activity Provided opportunities for critical thinking and reflection Promoted research leadership and research capacity within the health district/ organisation
Research Education Intervention [68]	Pre-and post- program surveys (Edmonton Research Orientation Survey – EROS Tool) at 3 time points; research proposal submission and summary of the feedback provided. *n =* 194 survey participants at 3 time points (control and intervention) *n =* 15 intervention participants	Attitudes and orientation to research (EROS) scores (at three time points)	Completion of a research proposal by intervention participants	Program led to the completion of several research studies (at one site) Ongoing support and mentoring are required for novice researchers to complete a research output An inverse association between higher EROS scores (i.e., a more positive orientation towards research) and no research activity, indicating that other factors were more influential on research activity Novice researchers working in teams were more likely to produce a research proposal
Qualitative Research Methodology and Methods (QRM) Training Program [82]	Ethnographic study (observations and interviews) *n =* 15 (including facilitator)	Educational, motivational, group-related, and organizational factors influencing skill acquisition and attitudes toward QRM	N/A	Experiential learning was effective in shifting participants’ mindsets about qualitative methodology Barriers to conduct qualitative research were related to time and the reputation qualitative research has among healthcare professionals Participants completed research projects which were shared with colleagues and managers
Writing for Publication Program [9]	Action research that drew of pre- and post-program surveys, post-workshop focus groups and facilitator reflections *n =* 9	Self-reported changes to writing for publication skills	Manuscript completion/ submission	Participants reported improved writing-for-publication Two submitted manuscripts to peer-reviewed journals Factors enabling manuscript completion were protected writing time, accountability to the mentors and clear, appropriate program timelines
Structure Professional Writing Retreat [32]	Survey and quantification of research outputs. *n =* 10 writing groups (4–8 nurses in each)	Research outputs: manuscripts submitted for publication, conference presentations, development of projects	Participant evaluation data on writing retreat purpose and objectives, consultants’ performance, and learning environment	Program led to the development of 9 manuscripts submitted for publication (4 accepted), other research outputs (oral and poster presentations) and the development of 2 nurse-led studies Mentors were integral to the progress of participants’ manuscripts
Practice-Based Research Challenge [64]	Survey *n =* 14	Perceived benefits of program	Perceived challenges associated with program participation	Participants reported gaining research knowledge, skills, and experience, professional development, and improving patient care Challenges related to the time commitment required of the program, recruiting research participants, and analysing data Access to a research mentor was considered a key enabler
Hope Research Community of Practice [86]	Interviews *n =* 5	Participants’ confidence and competence to complete a research project	Essential component parts of building a community of practice Project completion Supports and challenges that influenced project completion	There were three withdrawals from the program and four that were sustained Participants described feeling more competent and confident in their ability to conduct research Challenges to nurses engaging in research remain despite engagement in the HRCoP
*i*CAHE Journal Club [70]	Pre- and post- EBP surveys (Adapted Fresno Test and EBP Uptake) 12 JCs *n =* 93	Objective EBP knowledge and skills	Self-reported EBP uptake (behaviours, attitudes to, and perceived knowledge of EBP)	Program led to increases in EBP knowledge and behaviour outcomes across the allied health professions, with some showing more consistent improvements across the domains (physiotherapy) JCs are an effective teaching method that can incorporate principles of adult learning Barriers to research uptake were addressed through the collaborative learning between *i*CAHE researchers and JC clinicians
Clinical Nurse Research Fellows Program [92]	Informal measure of program outcomes *n =* 6	Research outputs (successful grants, grant applications, practice change, and subsequent research)	N/A	One nurse fellow was awarded a research grant, and another was encouraged to apply One project formed the foundation of a multicentre study, and two others were expected to inform health practice change The program was resource intensive
Nursing Research Fellowship [83]	Records of ethical approval of research projects and research dissemination *n =* 18	Number of projects with ethics approval	Research dissemination (via conferences and manuscripts) Grant funding awarded Cost of fellowships	Participants/ fellows each established a research project Research was disseminated internally by almost all participants; more than half presented externally and Twenty-one manuscripts were submitted
Research Education Lecture Series [53]	Retrospective pre-and six-month post-program survey *n =* 49	Self-reported research experience: writing a protocol, qualitative and quantitative research methods, publishing research, writing a research report, analysing and interpreting data, generating research ideas, applying for research funding	Intent to become involved in research: applying for research funding, analysing qualitative or quantitative data, writing a research protocol, writing a literature review, submitting an ethics application, writing for publication, collecting data Current involvement in research	Increased self-reported experience and engagement in research six months post-training Increased intent to become involved in research Increased experience and intent to engage in a wide range of research activities were reported (e.g., protocol development, ethics application, research activity, funding submissions) Training acted as a catalyst for participant with pre-existing interest in research to initiate research
Tailored EBP Education [56]	Pre- and post-training surveys (Evidence-based Practice Confidence scale, adapted Fresno test, and adapted EBP Implementation Scale) and focus groups *n =* 16 (surveys) *n =* 7 (focus groups)	EBP self-efficacy, knowledge (objective) and skills	Integration of learnings into practice (self-reported behaviour change)	Tailored education was deemed to be feasible for clinicians to participate in, and led to improvements in self-reported EBP self-efficacy and behaviour Increased EBP knowledge and skills were evident across the five EBP steps Self-reported EBP behaviour change was sustained three months post-training
EBP Education and Mentoring Program [75]	Pre- and post-program EBP Questionnaire (EBPQ) *n =* 9	Self-reported EBP knowledge and skills, practice, and attitudes towards EBP		Total EBP scores increased after the program, with the largest increases seen in the EBP knowledge and skills domain Participants attitudes did not improve significantly after the program which is likely due to their already positive attitudes EBP changes were observed, although it was recognised that the program is resource intensive Manager support for the program was key to its success
Course on Research Skills (Pilot) [45]	Course evaluation survey *n =* 69	Effectiveness of sessions	Intentions to change practice (qualitative)	Participants reported high effectiveness of the sessions and provided positive feedback on their experiences Participants anticipated changes to their practice as a result of the program
EBP and Research Utilization Education [65]	Single blind RCT Pre- and post-intervention Evidence-Based Readiness Inventory survey (baseline, 1-week post, 8-weeks post, and 4-months post) *n =* 43 (intervention) *n =* 34 (control)	EBP confidence / self-efficacy	EBP knowledge (objective)	Both the intervention and control groups demonstrated increased confidence in EBP and objective EBP knowledge after participation in the education program
Rural Research Capacity Building Program [85]	Pre- and post- survey (Research Spider tool) *n =* 130	Self-reported research experience across 10 research domains	N/A	Increased self-reported experience across all 10 research domains Greatest change in research protocol development and report-writing which aligned with components of the training
Centre for Research Excellence Rural Research Capacity Building Program [93]	Pre- and post- survey (Research Spider tool) Evaluation survey *n =* 7 (trainees) *n =* 4 (managers) *n =* 8 (facilitators)	Self-reported outcomes	Self-reported research experience across 10 research domains	Two trainees completed research reports at the end of the 2-year program, 4 presented research in a scientific forum and several had progressed their manuscripts The combination of education, mentoring, manager, and workplace support enabled trainees to persevere with their research Participants’ self-reported research experience improved after the program
PEAK (Physical therapist driven Education for Actionable Knowledge translation) Program [72, 102]	Pre- and post-surveys (EBP Beliefs Scale, Evidence-based Practice Confidence Scale, modified Fresno test, and the EBP Implementation Scale, self-reported participation in EBP) Interviews/focus groups *n =* 18	EBP self-efficacy, knowledge (objective) and skills	Self-reported behaviour change	Program improved EBP self-efficacy and self-reported behaviours Collaborative nature of the program was considered particularly valuable Additional support is needed to enhance knowledge and skills related to statistics
Systematic Review Workshop [76]	Researchers’ observations (action research) Outputs produced	Challenges and enablers	Publications	233 participants produced 414 research questions, and approximately one third of participants completed the workshop 13 peer-reviewed articles were published as a result of the workshop (a 3-fold increase on prior to workshops) Lack of time due to competing clinical demands was a common reason for program non-completion
TREAT (Tailoring Research Evidence and Theory) Journal Clubs [69]	A cluster RCT with nested focus group for intervention group Pre- and post-intervention EBP survey with additional items measuring satisfaction *n =* 41 (survey: intervention) *n =* 39 (survey: control) *n =* 8 (focus group)	EBP practice, attitudes, and knowledge of EBP (EBP questionnaire)	Competence in EBP (Assessing competence in evidence-based practice [ACE] tool) Clinician experiences of journal clubs (focus groups) Satisfaction with program Self-reported changes to clinical practice	EBP skills were maintained in participants of both journal club formats TREAT journal club participants were more satisfied with the format than those in the control group TREAT journal club participants valued the presence of an academic facilitator, the collaborative approach to critical appraisal, and structured tools to guide journal paper appraisal Standard JC participants made more changes to practice than TREAT participants Delivery of a structured, evidence-based journal club was deemed feasible
Hybrid Model Journal Club [54]	Pre- and post- program survey and evaluation survey *n =* 26 (EBP survey) *n =* 21 (evaluation survey)	EBP use	Satisfaction with program Attendance	Slight improvements in EBP use and behaviours Participants in both modes (in-person and online) were satisfied with the program Attendance was more consistent for the in-person group
Training and Mentoring Program [84]	Pre- and post-program surveys Qualitative interviews *n =* 21 (1-year surveys) *n =* 6 (2-year follow-up surveys) *n =* not stated for interviews	Surveys Research knowledge, confidence, behaviour, utilization, satisfaction, sustainability	Interviews Self-reported impact of research findings on practice Dissemination (in-service, conferences, posters) Facilitators for learning Barriers to learning Barriers to research (thematic analysis)	Program extended participants’ knowledge, skills and confidence in evaluation and research Participants applied the learnings directly to locally relevant research topics Role of mentorship (formal and informal) across the life of the project was found to be critical Peer relationships influenced participants’ commitment to completing projects

Most programs were evaluated using surveys (*n =* 51), some of these in combination with other outcome measures. More than half of the program evaluations (*n =* 38) used pre- and post-intervention surveys. Other evaluation methods included interviews, focus groups, attendance rates, and outcomes audits (e.g., ethics applications, manuscripts submitted for peer review or published, grant applications, grants awarded, or adherence to evidence-based guidelines). Twelve evaluation studies included a control group [36, 38, 51, 60, 65, 68–70, 77, 79, 86, 100]. Three evaluations were informal and did not explicitly draw on evaluation data but rather on general feedback, authors’ own reflections and observations, including observed research progress [35, 37, 94]. Evaluation of the longer-term outcomes were described in seven papers, where surveys were undertaken or outcomes were otherwise measured between one and 5 years after the programs were completed [44, 51, 76, 84, 85, 89, 93].

### Outcomes measured and described

Program outcome measures were mapped to Barr et al.’s modified Kirkpatrick educational outcomes typology [27]. The typology categorises educational outcomes reported according to their level of impact. The outcomes levels range from individual learner-level outcomes through to the impact of educational program on their organisation and healthcare consumer outcomes. See Table 4 below for descriptions of the outcome levels and the corresponding citations.

**Table 4:** Evaluation outcomes according to Barr et al.’s modified Kirkpatrick typology

Level and label	Description	Citations
1: Reaction	Participants’ experiences of, or satisfaction with the research education program	[29, 32–34, 39–45, 47, 49, 51, 52, 54, 57, 58, 61, 66, 69, 73, 74, 78, 84, 87, 91]
2a: Attitudes toward research	Participants’ self-reported changes in research attitudes	[33, 36, 40, 41, 43, 45, 46, 55, 62, 68, 69, 71, 75, 80, 89, 100, 102]
2b: Knowledge, skills, or confidence	Self-reported changes in research knowledge, skills, or confidence Objective measures of research knowledge or skills	[9, 31, 36–44, 46, 47, 50–53, 55, 56, 58, 59, 61, 63–65, 67, 69–71, 75, 79, 81, 82, 84, 86, 87, 89–91, 93, 102] [30, 38, 48, 56, 63, 65, 66, 69–71, 102]
3: Behaviour	Self-reported changes in research activity / behaviour Observed behaviours / research outputs (e.g., protocols, manuscripts, conference presentations, grants)	[30, 34, 38, 53, 54, 56, 62, 66, 69, 71, 74, 75, 77, 80, 81, 84, 85, 88, 93, 100, 102] [31, 34, 35, 46, 48, 59, 64, 73, 74, 78, 87, 90–92, 94]
4a: Organisational culture or practice	Changes in organisational research culture or practice (e.g., new research infrastructure, networks, or impact on program participants’ colleagues / teams)	[37, 38, 50, 55, 58, 60, 69, 79, 90, 100]
4b: Health consumer outcomes	None reported	Not applicable

Almost all program evaluations included a mix of outcome measure types or levels. In addition to the modified Kirkpatrick level outcomes, other types of outcomes and impacts were measured and reported. Program participant engagement was measured and reported with reference to interest and uptake, attendance, and drop-out rates in five evaluations [48, 54, 74, 78, 87]. Twelve program evaluations explored participants’ experiences or perspectives of barriers to engaging in research in their health setting [34, 36, 49, 56, 71, 77, 81, 82, 84, 86, 88, 89] and four evaluations included program cost calculations [51, 60, 83, 90]. One evaluation measured group cohesion, participant (nurse) productivity and nursing staff retention [100].

Programs that were evaluated over a longer period demonstrated a high success rate with respect to manuscript publication [34, 51, 76], longer term development of research skills, experience, and engagement [44, 84, 89], and highlighted the value of mentoring to participants’ enduring engagement with research and to their development of research confidence and leadership skills [84]. One evaluation study included administrative leaders [89], one included training participants’ managers [93], however none included senior executives or healthcare consumers.

## Discussion

To the authors’ knowledge, this is the first systematic scoping review of the research education literature. The findings of the review support existing evidence of the continued relevance of research education and training to RCB endeavours [2, 16]. Indeed, research education appears to be a mainstay RCB strategy over the last five decades. This review sought to explore the features or characteristics of research education and training programs delivered to nurses and allied health professionals working in health settings in HICs, the pedagogical principles or learning theories underpinning the programs, how programs were evaluated, and the types of outcomes reported.

Common features and approaches to the delivery of research education were identified. Some common pedagogical features of research education programs: multifaceted delivery to allow for flexibility in engaging with the program and content [5, 103], experiential learning [2, 103] and social or collaborative learning principles [103]. These underpinning principles were implied more frequently than they were explicitly stated. The integration of mentoring to reinforce the knowledge gleaned through research education programs appears to be a critical element and a key component of contemporary research education and capacity building [2, 3, 104].

This review also highlights some differences in the programs, particularly in terms of duration, which varied from single sessions or workshops to three-year programs. The curricula or educational content tended to reflect the aims of the programs which mapped to two different levels of engagement with research: research use or consumption and research activity. Some programs were specifically focused on advanced research skills, namely writing for publication, which is a particularly challenging aspect of the research process for clinicians [7, 51].

Findings indicate that organisational context and support are pivotal to the cultivation of and completion of research activity [2, 6, 7, 49, 77, 84, 88, 105]. Although this review focused specifically on papers describing research education programs targeting individual-level research capacity, there were several organisation-related factors that were integrated into the programs. Middle or executive level manager support for program participants was evident in numerous papers either through explicit support or permission, or positive role modelling. This resonates with the findings of existing evidence related to organisational factors enabling research [7, 106, 107]. Schmidt and colleagues [106] have previously highlighted a lack of managerial support for research training participants and their projects, as a factor influencing withdrawal. Several programs incorporated events or other opportunities for participants to present their work or to be otherwise recognised [37, 46, 54, 66, 80–83]. This facilitated organisation-level acknowledgement and celebration of individuals’ research activity and achievement, reinforcing organisational support for research [2].

This scoping review highlights some evidence of the impact of research education beyond the individual participants, and on their colleagues and organisations more broadly. This broader impact can be attributed to participants actively sharing their new knowledge and skills with their colleagues and teams [108]. Roger’s Diffusion of Innovation Theory can also underpin RCB strategies that are targeted at the individual level and explain how and why they have a broader impact on organisational research capacity and culture [104].

Research education program outcome measures tend to reflect lower levels of Kirkpatrick’s modified typology, with comparatively few studies reporting organisation-level impacts and none reporting health consumer outcomes. Although it is recognised that measuring and demonstrating direct links between RCB initiatives and health consumer outcomes is difficult [109], RCB initiatives including research training typically aim to promote the delivery of evidence-informed care, which in turn improves health consumer outcomes [110]. Some program evaluations included self-reported measures by participants that did not engage in the research education program, providing for comparisons between groups. Senior and executive managers, and healthcare consumers, however, were not involved in any evaluations reported. This limits knowledge of the outcomes and impacts beyond the individual participant level. Moreover, the program evaluation methods were generally poorly described. This is somewhat paradoxical, given the subject matter, however it is not a problem unique to research education and capacity building. Indeed poor evaluation is a widespread problem evident in multiple key healthcare areas such as Aboriginal Health in Australia [111] supportive care services for vulnerable populations [112], and in continuing education for healthcare professionals [113]. Factors contributing to poor program evaluation likely include time constraints, inaccessible data, and inadequate evaluation capacity and skills, as described in other scoping reviews of health and health professions education programs [111–113].

Although it is encouraging to see broadening interest in RCB initiatives for the nursing and allied health professions including research education, investment in rigorous, carefully planned, broadly targeted and long-term evaluation is required. This will ensure that research education programs maximise the outcomes for individuals and organisations and the most crucial impact on health consumer outcomes can be measured.

### Strengths and methodological limitations

The strengths of this scoping review are the adherence to an established and systematic approach and the wide and comprehensive search including 11 research databases, multiple grey literature databases and search engines. The methodological and content expertise within the research team, including expertise in scoping review, systematic review, realist review methodologies and research education and capacity building strategies strengthened the rigour of the review. Moreover, the consultation with content experts during the development of the search strategy ensured the review was well-informed and shaped to meet the needs of those concerned with RCB.

Nonetheless, this review is limited by several factors. Research education, training, and RCB more broadly are poorly defined concepts [2], as such, it is acknowledged that the search strategy was developed in such a way that it may not have resulted in the retrieval of all relevant literature. This is acceptable, given the scoping review aimed to provide an overview of the breadth and depth of the literature and used content expertise to balance the comprehensiveness of the review with the capacity to answer research questions [114]. It is, however, recommended that the findings of this review inform a more focused and systematic review of the literature.

It is well-established that research education and training alone, do not sufficiently influence research capacity and capability at an individual or organisational level [1, 7]. Indeed, barriers to nurse and allied health-led research include time constraints, demanding clinical workloads, enduring workforce shortages, a lack of organisational support and research culture, funding, and inadequate research knowledge and skills, persist [7, 12, 39, 47, 115]. These factors were not analysed as part of the review. The explicit focus on research education meant that some RCB strategies with education as a component may have been missed.

The authorship team were situated in Australia, with limited knowledge of other, complementary search engines internationally and lacked the resources to execute extensive international grey literature searches. These limited grey literature searches introduce a level of publication bias. Publications in languages other than English were excluded for reasons related to feasibility and limited resourcing. Through engagement with content experts early in the review, it was noted that many education programs are not formally documented, evaluated, or published in peer-reviewed or grey literature and therefore not accessible to others outside the organisation. This means that the review of published literature may not entirely represent research education programs in health settings.

## Conclusion

Research education is a cornerstone RCB strategy for nurses and allied health professionals working in health settings. Education is typically aimed at enhancing individual clinician-level RCB however, there is some evidence that the outcomes of individual-level research education can influence organisational research capacity and culture. Moreover, strategies targeted at the organisational level can be integrated into research education programs. Mentoring, experiential, and collaborative learning have gained recognition as key features of research education programs and facilitate the application of new knowledge and skills in practice. Evaluation continues to focus on lower levels of educational impact or traditional research outputs; there is need for greater attention to organisational culture, longer-term capacity building outcomes and health consumer impacts. Approaches to the evaluation of research education programs should incorporate the experiences and perspectives of managers, executives, health consumers and other stakeholders concerned with research capacity and the delivery of evidence-informed care. This will ensure that RCB strategies and initiatives with greater impact at the individual and organisational level can be supported and that the impact of such initiatives can be measured at the population health level.

## Supplementary Information

**Additional file 1.**

**Additional file 2.**

**Additional file 3.**

**Additional file 4.**

**Additional file 5.**

## Data Availability

All data generated or analysed during this study are included in this published article and its supplementary information files.

## Abbreviations

EBP: Evidence based practice
JBI: Joanna Briggs Institute
HIC: High-income countries
OECD: Organisation for Economic Co-operation and Development
PCC: Population, Concept and Context
PRISMA: Preferred Reporting Items for Systematic reviews and Meta-Analyses
PRISMA-ScR: PRISMA extension for scoping reviews
RCB: Research capacity building
RCT: Randomised control trial
