# Supplementary material for: Research education and training for nurses and allied health professionals: a systematic scoping review
Source: BMC Med Educ. 2022 May 19;22:385. doi: 10.1186/s12909-022-03406-7 (PMC9121620; doi:10.1186/s12909-022-03406-7)
Supplement: Supplementary file 1 — Additional file 1. [file 12909_2022_3406_MOESM1_ESM.docx]

# Additional File 1 - Scoping review protocol

The review questions, objectives and inclusion/exclusion criteria were developed by the research team and in consultation with four stakeholders. These formed a protocol for undertaking a scoping review using the Joanna Briggs Institute’s Methodology for Scoping Reviews (1).

## Review aim

To scope the literature on research education and training programs delivered to nurses and allied health professionals working in health settings and the evidence supporting these approaches.

## Review questions

1. What types of research education programs are delivered in health settings in high-income countries?

2. What theoretical or pedagogical principles underly the programs?

3. How are research education programs evaluated?

4. What types of outcomes are reported?

#### Table 1. Inclusion and exclusion criteria

|  | **Inclusion criteria** | **Exclusion criteria** |
| --- | --- | --- |
| **Population** | Health professionals working in healthcare settings including nurses, midwives, allied health professionals (e.g., physiotherapists, dietitians, speech pathologists, social workers, occupational therapists, podiatrists, dietitians), pharmacists* | Medical doctors  Health professionals working in non-health / academic settings  Undergraduate students |
| **Concept** | Research capacity building/ development programs, research-orientated continuing education, in-services, training, workshops, workplace learning or mentorship  Evaluated programs† | Fellowships, scholarships, or other new roles (e.g., knowledge brokers, embedded researchers, librarian), research education as part of tertiary course, global research capacity building frameworks and programs |
| **Context** | Research or capacity building programs delivered in the healthcare setting in high-income countries (according to OECD criteria) (2) | Programs delivered in academic institutions and non-health settings or in low and middle-income countries |

Databases to be searched: PubMed, Ovid MEDLINE, Embase, CINAHL, VOCEDPlus, PEDro, Scopus, ERIC, Informit Health Database, JBI, and Google Scholar. Multiple platforms were searched for grey literature. See Tael 2 for example search strategy.

## Data extraction

Data extraction is to be presented in two tables categorized under the following headings:

(1) citation; name of program, country, and years of implementation; Education program: description and aims, Number of participants, profession/s, setting; Pedagogical principle or capacity-building theory

(2) Program; evaluation data collection method and sample size; primary outcome, secondary and other outcomes; key findings.

#### Table 2. Search strategy: Ovid MEDLINE

| **Search line** | **Query** | **Hits** |
| --- | --- | --- |
| 1 | exp Health Personnel/ | 574,246 |
| 2 | (health professional* or health staff or health worker* or practitioner* or clinician*).ab,ti. | 483,587 |
| 3 | nurs*.ab,ti. | 479,875 |
| 4 | midwi*.ab,ti. | 26,338 |
| 5 | (allied health or physiotherap* or dietitian* or dietician or (speech adj3 (therap* or patholog*)) or social work* or occupational therap* or podiatrist* or audiologist* or psychologist* or pharmacist* or paramedic*).ab,ti. | 144,100 |
| 6 | exp Health Services/ | 2,320,247 |
| 7 | exp Outpatients/ | 19,064 |
| 8 | (healthcare or health care or hospital* or in-hospital or community health or outpatient* or health service*).ab,ti. | 2,102,886 |
| 9 | or/1-8 | 4,551,212 |
| 10 | exp Capacity building/ | 3,161 |
| 11 | research.ab,ti. | 1,692,719 |
| 12 | Research Support as Topic/ | 22,903 |
| 13 | 11 or 12 | 1,704,717 |
| 14 | 10 and 13 | 1,303 |
| 15 | (research adj1 (capacity building or capacity development)).ab,ti. | 351 |
| 16 | (building research or promot* research or research* development or research enhancement).ab,ti. | 3,895 |
| 17 | research education.ab,ti. | 1,598 |
| 18 | ((education* program* or education* intervention or education* training or research training) and (research skill* or research method* or research appraisal* or research competenc* or utili?e research or research utili?ation or using research or conduct* research)).ab,ti. | 663 |
| 19 | ((course* or workshop* or seminar* or journal club* or participatory learning) and (research skill* or research method* or research appraisal* or research competenc* or utili?e research or research utili?ation or using research or conduct* research)).ab,ti. | 1,756 |
| 20 | (research and mentor* program*).ab,ti. | 607 |
| 21 | (scholarly writing or writing support or publication program*).ab,ti. | 141 |
| 22 | (research fellowship or research internship).ab,ti. | 346 |
| 23 | or/15-22 | 8,927 |
| 24 | 14 or 23 | 9,981 |
| 25 | 9 and 24 | 5,078 |
| 26 | limit 25 to english language | 4,864 |

1. Peters MD, Marnie C, Tricco AC, Pollock D, Munn Z, Alexander L, et al. Updated methodological guidance for the conduct of scoping reviews. JBI Evid Synth. 2020;18(10):2119-26.

2. The World Bank. Data for High income, OECD members, Upper middle income <https://data.worldbank.org/?locations=XD-OE-XT2021> [Available from: <https://data.worldbank.org/?locations=XD-OE-XT>.
