# Supplementary material for: Research education and training for nurses and allied health professionals: a systematic scoping review
Source: BMC Med Educ. 2022 May 19;22:385. doi: 10.1186/s12909-022-03406-7 (PMC9121620; doi:10.1186/s12909-022-03406-7)
Supplement: Supplementary file 3 — Additional file 3. [file 12909_2022_3406_MOESM3_ESM.docx]

# Additional File 3 Electronic search terms and results

**Database: Medline via Ovid**

**Date: 12/3/2022**

| **Search line** | **Query** | **Hits** |
| --- | --- | --- |
| 1 | exp Health Personnel/ | 574,246 |
| 2 | (health professional* or health staff or health worker* or practitioner* or clinician*).ab,ti. | 483,587 |
| 3 | nurs*.ab,ti. | 479,875 |
| 4 | midwi*.ab,ti. | 26,338 |
| 5 | (allied health or physiotherap* or dietitian* or dietician or (speech adj3 (therap* or patholog*)) or social work* or occupational therap* or podiatrist* or audiologist* or psychologist* or pharmacist* or paramedic*).ab,ti. | 144,100 |
| 6 | exp Health Services/ | 2,320,247 |
| 7 | exp Outpatients/ | 19,064 |
| 8 | (healthcare or health care or hospital* or in-hospital or community health or outpatient* or health service*).ab,ti. | 2,102,886 |
| 9 | or/1-8 | 4,551,212 |
| 10 | exp Capacity building/ | 3,161 |
| 11 | research.ab,ti. | 1,692,719 |
| 12 | Research Support as Topic/ | 22,903 |
| 13 | 11 or 12 | 1,704,717 |
| 14 | 10 and 13 | 1,303 |
| 15 | (research adj1 (capacity building or capacity development)).ab,ti. | 351 |
| 16 | (building research or promot* research or research* development or research enhancement).ab,ti. | 3,895 |
| 17 | research education.ab,ti. | 1,598 |
| 18 | ((education* program* or education* intervention or education* training or research training) and (research skill* or research method* or research appraisal* or research competenc* or utili?e research or research utili?ation or using research or conduct* research)).ab,ti. | 663 |
| 19 | ((course* or workshop* or seminar* or journal club* or participatory learning) and (research skill* or research method* or research appraisal* or research competenc* or utili?e research or research utili?ation or using research or conduct* research)).ab,ti. | 1,756 |
| 20 | (research and mentor* program*).ab,ti. | 607 |
| 21 | (scholarly writing or writing support or publication program*).ab,ti. | 141 |
| 22 | (research fellowship or research internship).ab,ti. | 346 |
| 23 | or/15-22 | 8,927 |
| 24 | 14 or 23 | 9,981 |
| 25 | 9 and 24 | 5,078 |
| 26 | limit 25 to english language | 4,864 |

**Database: CINAHL**

**Date: 12/3/2022**

| **Search line** | **Search Terms** | **Hits** |
| --- | --- | --- |
| **S1** | (MH "Health Personnel+") | 622,566 |
| **S2** | TI “health professional*” OR AB “health professional*” OR TI “health staff” OR AB “health staff” OR TI “health worker*” OR AB “health worker*” OR TI practitioner* OR AB practitioner* OR TI clinician* OR AB clinician* | 262,505 |
| **S3** | TI nurs* OR AB nurs* | 601,151 |
| **S4** | TI midwi* OR AB midwi* | 38,195 |
| **S5** | TI “allied health” OR AB “allied health” OR TI physiotherap* OR AB physiotherap* OR TI dietitian* OR AB dietitian* OR TI dietician* OR AB dietician* OR (TI speech N3 (TI therap* OR TI patholog*)) OR (AB speech N3 (AB therap* OR AB patholog*)) OR TI “social work*” OR AB “social work*” OR TI “occupational therap*” OR AB “occupational therap*” OR TI podiatrist* OR AB podiatrist* OR TI audiologist* OR AB audiologist* OR TI psychologist* OR AB psychologist* OR TI pharmacist* OR AB pharmacist* OR TI paramedic* OR AB paramedic* | 134,612 |
| **S6** | (MH "Health Services+") | 1,116,518 |
| **S7** | (MM "Outpatients") | 2,168 |
| **S8** | TI healthcare OR AB healthcare OR TI “health care” OR AB “health care” OR TI hospital* OR AB hospital* OR TI in-hospital OR AB in-hospital OR TI “community health” OR AB “community health” or TI outpatient* OR AB outpatient* OR TI “health service*” OR AB “health service*” | 947,080 |
| **S9** | S1 OR S2 OR S3 OR S4 OR S5 OR S6 OR S7 OR S8 | 2,545,817 |
| **S10** | TI research N1 (TI “capacity building” OR TI “capacity development”) OR AB research N1 (AB "capacity building" OR AB "capacity development") | 315 |
| **S11** | TI “building research” OR AB “building research” OR TI “promot* research” OR AB “promot* research” OR TI “research* development” OR AB “research* development” OR TI “research enhancement” OR AB research enhancement” | 2,133 |
| **S12** | TI “research education” OR AB “research education” | 1,147 |
| **S13** | (TI “education* program*” OR AB “education* program*” OR TI “education* intervention” OR AB “education* intervention” OR TI “education* training” OR AB “education* training” OR TI “research training” OR AB “research training”) AND (TI “research skill*” OR AB “research skill*” OR TI “research method*” OR AB “research method*” OR TI “research appraisal*” OR AB “research appraisal*” OR TI “research competenc*” OR AB “research competenc*” OR TI “utili#e research” OR AB “utili#e research” OR TI “research utili#ation” OR AB “research utili#ation” OR TI “using research” OR AB “using research” OR TI “conduct* research” OR AB “conduct* research”) | 476 |
| **S14** | (TI course* OR AB course* OR TI workshop* OR AB workshop* OR TI seminar* OR AB seminar* OR TI “journal club*” OR AB “journal club*” OR TI “participatory learning” OR AB “participatory learning”) AND (TI “research skill*” OR AB “research skill*” OR TI “research method*” OR AB “research method*” OR TI “research appraisal*” OR AB “research appraisal*” OR TI “research competenc*” OR AB “research competenc*” OR TI “utili#e research” OR AB “utili#e research” OR TI “research utili#ation” OR AB “research utili#ation” OR TI “using research” OR AB “using research” OR TI “conduct* research” OR AB “conduct* research”) | 1,175 |
| **S15** | (TI research AND TI “mentor* program*”) OR (AB research AND AB “mentor* program*”) | 464 |
| **S16** | TI “scholarly writing” OR AB “scholarly writing” OR TI “writing support” OR AB “writing support” OR TI “publication program*” OR AB “publication program*” | 164 |
| **S17** | TI “research fellowship” OR AB “research fellowship” OR TI “research internship” OR AB “research internship” | 184 |
| **S18** | S10 OR S11 OR S12 OR S13 OR S14 OR S15 OR S16 OR S17 | 5,758 |
| **S19** | S9 AND S18 | 3,666 |
| **S20** | Narrow by Language: - english | 3,523 |

Note: Capacity building does not exist as subject heading thus it and related concepts were omitted.

| **Database: Embase**  **Date: 12/3/2022**   \| **Search line** \| **Search Terms** \| **Hits** \| \| --- \| --- \| --- \| \| **#1** \| 'health care personnel'/exp \| 1,792,882 \| \| **#2** \| ('health professional* OR 'health staff' OR 'health worker* OR practitioner* OR clinician*):ab,ti \| 671,170 \| \| **#3** \| nurs*:ab,ti \| 589,654 \| \| **#4** \| midwi*:ab,ti \| 31,414 \| \| **#5** \| 'allied health':ab,ti OR physiotherap*:ab,ti OR dietitian*:ab,ti OR dietician:ab,ti OR ((speech NEAR/3 (therap* OR patholog*)):ab,ti) OR 'social work*':ab,ti OR 'occupational therap*':ab,ti OR podiatrist*:ab,ti OR audiologist*:ab,ti OR psychologist*:ab,ti OR pharmacist*:ab,ti OR paramedic*:ab,ti \| 254,378 \| \| **#6** \| 'health service'/exp \| 6,362,198 \| \| **#7** \| 'outpatient'/exp \| 142,734 \| \| **#8** \| (healthcare OR 'health care' OR hospital* OR 'in-hospital' OR 'community health' OR outpatient* OR 'health service*'):ab,ti \| 3,232,008 \| \| **#9** \| #1 OR #2 OR #3 OR #4 OR #5 OR #6 OR #7 OR #8 \| 9,033,537 \| \| **#10** \| 'capacity building'/exp \| 6,107 \| \| **#11** \| research:ab,ti \| 2,313,437 \| \| **#12** \| 'research'/mj \| 122,534 \| \| **#13** \| #11 OR #12 \| 2,412,643 \| \| **#14** \| #10 AND #13 \| 2,337 \| \| **#15** \| (research NEAR/1 ('capacity building' OR 'capacity development')):ab,ti \| 417 \| \| **#16** \| ('building research' OR 'promot* research' OR 'research* development' OR 'research enhancement'):ab,ti \| 5,430 \| \| **#17** \| 'research education':ab,ti \| 2,160 \| \| **#18** \| ('education* program*' OR ‘education* intervention’ OR 'education* training' OR ‘research training’):ab,ti AND ('research skill*’ OR ‘research method*’ OR ‘research appraisal*’ OR 'research competenc*' OR 'utilise research' OR ‘utilize research’ OR 'research utilisation' OR ‘research utilization’ OR ‘using research’ OR 'conduct* research'):ab,ti \| 974 \| \| **#19** \| (course* OR workshop* OR seminar* OR ‘journal club*’ OR ‘participatory learning’):ab,ti AND ('research skill*’ OR ‘research method*’ OR ‘research appraisal*’ OR 'research competenc*' OR 'utilise research' OR ‘utilize research’ OR 'research utilisation' OR ‘research utilization’ OR ‘using research’ OR 'conduct* research'):ab,ti \| 2,864 \| \| **#20** \| (research):ab,ti AND ('mentor* program*'):ab,ti \| 859 \| \| **#21** \| ('scholarly writing' OR 'writing support' OR 'publication program*'):ab,ti \| 575 \| \| **#22** \| ('research fellowship' OR 'research internship'):ab,ti \| 623 \| \| **#23** \| #15 OR #16 OR #17 OR #18 OR #19 OR #20 OR #21 OR #22 \| 13,269 \| \| **#24** \| #14 OR #23 \| 15,245 \| \| **#25** \| #9 AND #24 \| 8,887 \| \| **#26** \| #9 AND #24 AND [english]/lim \| 8,533 \|   **Database: PubMed**  **Date: 12/3/2022**   \| **Search line** \| **Search Terms** \| **Hits** \| \| --- \| --- \| --- \| \| **#1** \| health personnel [MeSH Terms] \| 574,882 \| \| **#2** \| “health professional”[Title/Abstract] OR “health professionals”[Title/Abstract] OR “health staff”[Title/Abstract] OR “health worker”[Title/Abstract] OR “health workers”[Title/Abstract] OR practitioner*[Title/Abstract] OR clinician*[Title/Abstract] \| 498,785 \| \| **#3** \| nurs*[Title/Abstract] \| 501,437 \| \| **#4** \| midwi*[Title/Abstract] \| 27,938 \| \| **#5** \| allied health[Title/Abstract] OR physiotherap*[Title/Abstract] OR dietitian*[Title/Abstract] OR dietician*[Title/Abstract] OR therap*[Title/Abstract] OR patholog*[Title/Abstract] OR social work[Title/Abstract] OR podiatrist*[Title/Abstract] OR audiologist*[Title/Abstract] OR psychologist*[Title/Abstract] OR pharmacist*[Title/Abstract] OR paramedic*[Title/Abstract] \| 4,143,131 \| \| **#6** \| health services [MeSH Terms] \| 2,322,402 \| \| **#7** \| outpatients [MeSH Terms] \| 19,103 \| \| **#8** \| healthcare[Title/Abstract] OR “health care”[Title/Abstract] OR hospital*[Title/Abstract] OR in-hospital[Title/Abstract] OR “community health”[Title/Abstract] OR outpatient*[Title/Abstract] OR “health service”[Title/Abstract] OR “health services”[Title/Abstract] \| 2,213,366 \| \| **#9** \| #1 OR #2 OR #3 OR #4 OR #5 OR #6 OR #7 OR #8 \| 8,046,356 \| \| **#10** \| capacity building [MeSH Terms] \| 3,164 \| \| **#11** \| research[Title/Abstract] \| 1,813,965 \| \| **#12** \| research support as topic [MeSH Terms] \| 22,906 \| \| **#13** \| #11 OR #12 \| 1,825,307 \| \| **#14** \| #10 AND #13 \| 1,329 \| \| **#15** \| “research capacity building”[Title/Abstract] OR “research capacity development”[Title/Abstract] \| 378 \| \| **#16** \| “building research”[Title/Abstract] OR “promote research”[Title/Abstract] OR “promoting research”[Title/Abstract] OR “researcher development”[Title/Abstract] OR “research development”[Title/Abstract] OR “research enhancement”[Title/Abstract] \| 2,837 \| \| **#17** \| “research education”[Title/Abstract] \| 1,712 \| \| **#18** \| ("education program"[Title/Abstract] OR "education programme"[Title/Abstract] OR “educational program”[Title/Abstract] OR “educational programme”[Title/Abstract] OR "education intervention"[Title/Abstract] OR “educational intervention”[Title/Abstract] OR "education training"[Title/Abstract] OR “educational training”[Title/Abstract] OR "research training"[Title/Abstract]) AND ("research skill”[Title/Abstract] OR “research skills"[Title/Abstract] OR “research methods”[Title/Abstract] OR “research methodology”[Title/Abstract] OR "research appraisal"[Title/Abstract] OR “research appraisals”[Title/Abstract] OR "research competency"[Title/Abstract] OR “research competencies”[Title/Abstract] OR "utilise research"[Title/Abstract] OR “utilize research”[Title/Abstract] OR "research utilisation"[Title/Abstract] OR "research utilization"[Title/Abstract] OR "conduct research"[Title/Abstract] OR “conducting research”[Title/Abstract]) \| 805 \| \| **#19** \| (course*[Title/Abstract] OR workshop*[Title/Abstract] OR seminar*[Title/Abstract] OR “journal club”[Title/Abstract] OR “participatory learning”[Title/Abstract]) AND ("research skill”[Title/Abstract] OR “research skills"[Title/Abstract] OR “research methods”[Title/Abstract] OR “research methodology”[Title/Abstract] OR "research appraisal"[Title/Abstract] OR “research appraisals”[Title/Abstract] OR "research competency"[Title/Abstract] OR “research competencies”[Title/Abstract] OR "utilise research"[Title/Abstract] OR “utilize research”[Title/Abstract] OR "research utilisation"[Title/Abstract] OR "research utilization"[Title/Abstract] OR "conduct research"[Title/Abstract] OR “conducting research”[Title/Abstract]) \| 2,785 \| \| **#20** \| (research[Title/Abstract] AND “mentoring program”[Title/Abstract]) OR (research[Title/Abstract] AND “mentor program”[Title/Abstract]) OR (research[Title/Abstract] AND “mentor programme”[Title/Abstract]) OR (research[Title/Abstract] AND “mentoring programme”[Title/Abstract]) \| 281 \| \| **#21** \| “scholarly writing”[Title/Abstract] OR “writing support”[Title/Abstract] OR “publication program”[Title/Abstract] OR “publication programme”[Title/Abstract] \| 157 \| \| **#22** \| “research fellowship”[Title/Abstract] OR “research internship”[Title/Abstract] \| 388 \| \| **#23** \| #15 OR #16 OR #17 OR #18 OR #19 OR #20 OR #21 OR #22 \| 8,932 \| \| **#24** \| #14 OR #23 \| 10,013 \| \| **#25** \| #9 AND #24 \| 5,612 \| \| **#26** \| Search: #9 AND #24 Filters: English \| **5,315** \| |  |  |  |  |
| --- | --- | --- | --- | --- | --- | --- | --- | --- | --- | --- | --- | --- | --- | --- | --- | --- | --- | --- | --- | --- | --- | --- | --- | --- | --- | --- | --- | --- | --- | --- | --- | --- | --- | --- | --- | --- | --- | --- | --- | --- | --- | --- | --- | --- | --- | --- | --- | --- | --- | --- | --- | --- | --- | --- | --- | --- | --- | --- | --- | --- | --- | --- | --- | --- | --- | --- | --- | --- | --- | --- | --- | --- | --- | --- | --- | --- | --- | --- | --- | --- | --- | --- | --- | --- | --- | --- | --- | --- | --- | --- | --- | --- | --- | --- | --- | --- | --- | --- | --- | --- | --- | --- | --- | --- | --- | --- | --- | --- | --- | --- | --- | --- | --- | --- | --- | --- | --- | --- | --- | --- | --- | --- | --- | --- | --- | --- | --- | --- | --- | --- | --- | --- | --- | --- | --- | --- | --- | --- | --- | --- | --- | --- | --- | --- | --- | --- | --- | --- | --- | --- | --- | --- | --- | --- | --- | --- | --- | --- | --- | --- | --- | --- | --- | --- | --- | --- |

Note: PubMed does not allow for proximity searching or for truncation and phrase searching to be combined. The phrase “using research” was omitted as the word “using” was not permitted by PubMed (stop word).

| **Database: Scopus**  **Date: 15/3/2022**  ((health professional* OR practitioner* OR clinician* OR nurs* OR allied health OR healthcare OR health care OR health service*).ab,ti. AND (research capacity OR research development OR research education* OR research training)).ab,ti. **65**  **Database: JBI**  **Date: 15/3/2022**  (health professional* OR practitioner* OR clinician* OR nurs* OR allied health OR healthcare OR health care OR health service*).ab,ti. AND (research capacity OR research development OR research education* OR research training).ab,ti. **2**  **Database: Informit**  **Date: 15/3/2022**  [Abstract: 'health professional' OR Abstract: practitioner* OR Abstract: clinician* OR Abstract: nurs* OR Abstract: 'allied health' OR Abstract: healthcare OR Abstract: 'health care' OR Abstract: 'health service'] AND [Abstract: 'research capacity' OR Abstract: 'research development' OR Abstract: 'research education' OR Abstract: 'research training'] **78**  **Database: ERIC**  **Date: 15/3/2022**  (AB "research capacity" OR AB "research development") AND (AB "health") **65**  **Database: VOCEDPlus**  **Date: 15/3/2022**   1. Abstract:("research capacity") AND Abstract:("health") **10** 2. Abstract:(“research development”) AND Abstract:(“health”) **1**   **Database: PEDro**  **Date: 15/3/2022**   1. “research capacity” in Abstract and Title **0** 2. “research development” in Abstract and Title **0**   **Google Scholar 17/3/2022**  **Date:**  1. Research capacity building nursing **200**  2. Research capacity building allied health **200**  3. Research skill development AND (nurse OR allied health OR clinician OR practitioner) **200**  After de-duplicating: **516** |  |  |  |  |
| --- | --- | --- | --- | --- |
