# Supplementary material for: Research education and training for nurses and allied health professionals: a systematic scoping review
Source: BMC Med Educ. 2022 May 19;22:385. doi: 10.1186/s12909-022-03406-7 (PMC9121620; doi:10.1186/s12909-022-03406-7)
Supplement: Supplementary file 4 — Additional file 4. [file 12909_2022_3406_MOESM4_ESM.docx]

# Additional file 4 Grey literature searches

| **Information source (e.g. name of organisation)** | **Website (url)** | **Search terms used and n results retrieved** | **Date of search** | **Citations retrieved** | **Name of citations** | **URL** |
| --- | --- | --- | --- | --- | --- | --- |
| Google | http://www.google.com | Research education health (n = 154,000,00) | 17/03/2022 | 2 | RETProgram  Research education \| South Eastern Sydney Local Health District | <https://wahtn.org/platforms/research-education-training-program/>  <https://www.seslhd.health.nsw.gov.au/services-clinics/directory/research-home/research-education> |
| Google | http://www.google.com | Research capacity building health (n=12,000,000) | 17/03/2022 | 1 | Impact of a research capacity building initiative | [Impact of a Research Capacity Building Initiative: Qualitative Evaluation of HP Research Positions (health.qld.gov.au)](https://www.health.qld.gov.au/__data/assets/pdf_file/0032/498380/qerpart1-qualeval.pdf) |
| Google | http://www.google.com | Research education health professionals (n= 169,000,000) |  | 0 |  |  |
| Google Scholar | [www.scholar.google.com](http://www.scholar.google.com/) | Research education health professionals (n=4,050,000) | 17/03/2022 | 1 | Annual Research Report, Education and Research Support | <https://www.health.qld.gov.au/__data/assets/pdf_file/0018/1011429/2019-annual-research-report.pdf> |
| Queensland Health | [Home \| Queensland Health](https://www.health.qld.gov.au/) | Research education (n=14,566) | 17/03/2022 | 1 | Training and Education (Allied Health Translating Research into Practice) | <https://www.health.qld.gov.au/clinical-practice/database-tools/translating-research-into-practice-trip/translating-research-into-practice> |
| Department of Health, Victoria | [Health.vic \| health.vic.gov.au](https://www.health.vic.gov.au/) | Research education/support/ research (n=2634) | 17/03/2022 | 0 |  |  |
| HETI | [My Health Learning \| HETI (nsw.gov.au)](https://www.heti.nsw.gov.au/education-and-training/my-health-learning) | Research education (n=1108) | 17/03/2022 | 1 | Rural Research Capacity Building Program Information and Application Guidelines | <https://www.heti.nsw.gov.au/__data/assets/pdf_file/0003/671817/RRCBP-Information-and-Application-Guidelines-2022.pdf> |
| ACT Health | [Search \| Health (act.gov.au)](https://www.health.act.gov.au/search?search=research) | Research education (n=2221) | 17/03/2022 | 1 | Research, teaching and training | <https://www.health.act.gov.au/research/act-health-summit-research-teaching-and-training> |
| Tasmania Health | [Tasmanian Department of Health \| Tasmanian Department of Health](https://www.health.tas.gov.au/) | Research education (n=43) | 17/03/2022 | 1 | Development and Research | <https://www.health.tas.gov.au/career/home/nurses2/development_and_research> |
| Western Australia Health | [WA Health, Government of Western Australia](https://ww2.health.wa.gov.au/) | Research education (n=50) | 17/03/2022 | 1 | Research Education Program | <https://ww2.health.wa.gov.au/News/2019/Research-education-program> |
| Northern Territory Health | [Homepage - NT Health](https://health.nt.gov.au/) | Research education (n=2174) | 17/03/2022 | 1 | Health research | <https://health.nt.gov.au/data-and-research/nt-health-research> |
| South Australia Health | [Home \| SA Health](https://www.sahealth.sa.gov.au/wps/wcm/connect/public+content/sa+health+internet/home/home) | Research education (n=1431) | 17/03/2022 | 0 |  |  |
| Australian Government Department of Health | https://www.health.gov.au/ | Research education (n = 1330) | 17/03/2022 | 0 |  |  |
| Services for Australian Rural and Remote Allied Health | https://sarrah.org.au/ | Research education (n=25) | 17/03/2022 | 1 | [Designing](https://sarrah.org.au/cpd/online-education/sarrah-practice-redesign-series?highlight=WyJyZXNlYXJjaCIsImVkdWNhdGlvbiJd) and implementing successful AHA modes of care | [Designing and Implementing Successful AHA Models of Care - SARRAH](https://sarrah.org.au/cpd/online-education/online-courses/521-designing-and-implementing-successful-aha-models-of-care?highlight=WyJyZXNlYXJjaCIsImVkdWNhdGlvbiJd) |
| Services for Australian Rural and Remote Allied Health | https://sarrah.org.au/ | Research capacity (n=10) | 17/03/2022 | 0 |  |  |
| Healthcare Management Information Consortium | [Ovid - HMIC Database \| Wolters Kluwer](https://www.wolterskluwer.com/en/solutions/ovid/hmic-database-99?top=2&mid=3&bottom=7&subsection=10) | Research education (n=363) | 17/03/2022 | 0 |  |  |
| Open grey repository | [Grey Literature - OpenGrey and GreyNets Collection](http://www.greynet.org/opengreyrepository.html) | Research education (n=1242) | 17/03/2022 | 0 |  |  |
| Open grey repository | [Grey Literature - OpenGrey and GreyNets Collection](http://www.greynet.org/opengreyrepository.html) | Research capacity (n=150) | 17/03/2022 | 0 |  |  |
| Canada Health | [Health Canada - Canada.ca](https://www.canada.ca/en/health-canada.html) | Research education (n=367,544) | 17/03/2022 | 1 | Health research training platform | <https://cihr-irsc.gc.ca/e/52278.html> |
| NHS | [The NHS website - NHS (www.nhs.uk)](https://www.nhs.uk/) | Research education (n=4) | 17/03/2022 | 0 |  |  |
| NHS | [The NHS website - NHS (www.nhs.uk)](https://www.nhs.uk/) | Research capacity (n=260) | 17/03/2022 | 0 |  |  |
| Council for Allied Health Professions Research | [Council for Allied Health Professions Research \| Council for Allied Health Professions Research (csp.org.uk)](https://cahpr.csp.org.uk/) | Research education (n=157) | 17/03/2022 | 1 | CAHPR Cheshire & Merseyside event - First Steps in Research | [CAHPR Cheshire & Merseyside event - First Steps in Research \| Council for Allied Health Professions Research (csp.org.uk)](https://cahpr.csp.org.uk/news/2018-03-01-cahpr-cheshire-merseyside-event-first-steps-research) |
