# Supplementary material for: Research education and training for nurses and allied health professionals: a systematic scoping review
Source: BMC Med Educ. 2022 May 19;22:385. doi: 10.1186/s12909-022-03406-7 (PMC9121620; doi:10.1186/s12909-022-03406-7)
Supplement: Supplementary file 5 — Additional file 5. [file 12909_2022_3406_MOESM5_ESM.docx]

# Additional File 5 – Excluded Studies

| **Reference** | **Reason for exclusion** |
| --- | --- |
| Wilkinson SA, Hough J, Hinchliffe F. An evidence-based approach to influencing evidence-based practice in allied health. Journal of Allied Health. 2016 Mar 3;45(1):41-8. | Insufficient description of education intervention |
| Abramovitz L, Baggott C. A novel nurse research mentorship program to promote evidence based practice. In Paediatric Blood & Cancer 2010 Nov 1 (Vol. 55, No. 5, pp. 802-802). | Insufficient description of education intervention |
| Alderson L, Waite G, Chugh D, Sell D. Delivering digital learning packages to address allied health professionals skill gaps in research. Physiotherapy. 2022 Feb 1;114:e83. | Insufficient description of education intervention |
| Bauer-Horvath H. Teaching evidence-based practical skills in clinical training–a collection of quality-assurance measures for clinical instructors. Physiotherapy. 2016 Nov 1;102:e189. | Insufficient description of education intervention |
| Farrukh M, Nasir A. A day experience at Research Methodology Workshop held by PAME. JPMA. The Journal of the Pakistan Medical Association. 2014 Nov;64(11):1335-6. | Insufficient description of education intervention |
| Rhondali W, Nguyen LM, Peck M, Vallet F, Daneault S, Filbet M. Description of a teaching method for research education for palliative care healthcare professionals. Palliative & Supportive Care. 2015 Apr;13(2):249-54. | Insufficient description of education intervention |
| Spence, K. "O-142 A Clinical Neonatal Nursing Research Fellowship: Linking Research To Practice." (2014): A78-A78. | Insufficient description of education intervention |
| Boggs L, Whelan L, Segrt A, Lang T. Breaking barriers to research skills training and enabling access to those who need it the most. Transactions of The Royal Society of Tropical Medicine and Hygiene. 2019 Sep 16;113(S1). | Insufficient description of education intervention |
| Black AT, Ali S, Baumbusch J, McNamee K, Mackay M. Practice‐based nursing research: Evaluation of clinical and professional impacts from a research training programme. Journal of Clinical Nursing. 2019 Jul;28(13-14):2681-7. | Insufficient description of education intervention |
| Bradley, R, Booth-La-Force, C, Zwickey, H. T90/R90 Building Research across Inter-Disciplinary Gaps (BRIDG) Clinical Research Training Program in Complementary and Integrative Health. World Congress Integrative Medicine & Health 2017: Part one. 2017 | Insufficient description of education intervention |
| Zakaria N, AlMutairi A, Zakaria N. Qualitative Research Methodology in Healthcare Workshop. Computer Methods and Programs in Biomedicine. 2021 Jun 1;205:105994. | Insufficient description of education intervention |
| Clifford C, Murray S. Pre‐and post‐test evaluation of a project to facilitate research development in practice in a hospital setting. Journal of Advanced Nursing. 2001 Dec 15;36(5):685-95. | Insufficient description of education intervention |
| Janssen J, Hale L, Mirfin-Veitch B, Harland T. Stimulating research involvement in Clinical physiotherapists. Physiotherapy. 2011;97(S1):eS549. | Insufficient description of education intervention |
| Melder A, Turner T, Allen K, Georgiou A, Harris C. Evidence based practice capacity building-enabling the process of getting evidence to the point of care in allied health. JBI Evidence Implementation. 2011 Sep 1;9(3):315-6. | Insufficient description of education intervention |
| Cameron HE, Boreland FT, Morris JR, Lyle DM, Perkins DA, Magin PJ, Marshall MJ, Zwar NA. New South Wales and Australian Capital Territory Researcher Development Program 2005–07: modest investment, considerable outcomes. Australian Journal of Primary Health. 2013 Mar 12;19(1):59-67. | Insufficient description of education intervention |
| Dyas J, Moody L, Siriwardena AN. Improving research quality and health care in rural areas: the contribution of a Teaching Primary Care Trust: a case study. Quality in Primary care. 2005;13(4):183-90. | Insufficient description of education intervention |
| Eames S, Bennett S, Whitehead M, Fleming J, Low SO, Mickan S, Caldwell E. A pre‐post evaluation of a knowledge translation capacity‐building intervention. Australian Occupational Therapy Journal. 2018 Dec;65(6):479-93. | Insufficient description of education intervention |
| Feng N, Edwards JK, Owiti PO, Zhang GM, Vallejo ZV, Hann K, Zhou SS, Oo MM, Geoffroy EM, Ma C, Li T. Operational research capacity building through the Structured Operational Research Training Initiative (SORT-IT) in China: implementation, outcomes and challenges. Infectious diseases of poverty. 2021 Dec;10(1):1-4. | Insufficient description of education intervention |
| Fowler L, Gottschlich MM, Kagan RJ. Burn center journal club promotes clinical research, continuing education, and evidence-based practice. Journal of burn care & research. 2013 Mar 1;34(2):e92-8. | Insufficient description of education intervention |
| Gullick JG, West SH. Building research capacity and productivity among advanced practice nurses: an evaluation of the Community of Practice model. Journal of advanced nursing. 2016 Mar;72(3):605-19. | Insufficient description of education intervention |
| Jacques J. Establishing an Evidence-based Journal Club for Radiation Therapists: the WRCC Experience. Journal of Medical Imaging and Radiation Sciences. 2014 Jun 1;45(2):180-1. | Insufficient description of education intervention |
| Klimas J, Fernandes E, DeBeck K, Hayashi K, Milloy MJ, Kerr T, Cullen W, Wood E. Preliminary results and publication impact of a dedicated addiction clinician scientist research fellowship. Journal of addiction medicine. 2017 Jan;11(1):80. | Insufficient description of education intervention |
| Lee G, Metcalf S. Building research capacity: through a hospital-based clinical school of nursing. Nurse Education Today. 2009 Apr 1;29(3):350-6. | Insufficient description of education intervention |
| Larrabee JH, Sions J, Fanning M, Withrow ML, Ferretti A. Evaluation of a program to increase evidence-based practice change. JONA: The Journal of Nursing Administration. 2007 Jun 1;37(6):302-10. | Insufficient description of education intervention |
| McIntyre E, Brun L, Cameron H. Researcher development program of the primary health care research, evaluation and development strategy. Australian Journal of Primary Health. 2011 Apr 6;17(1):114-21. | Insufficient description of education intervention |
| McKee G, Codd M, Dempsey O, Gallagher P, Comiskey C. Describing the implementation of an innovative intervention and evaluating its effectiveness in increasing research capacity of advanced clinical nurses: using the consolidated framework for implementation research. BMC nursing. 2017 Dec;16(1):1-3. | Insufficient description of education intervention |
| Mengato, D., and M. Chiumente. "6ER-019 Introducing young hospital pharmacists to scientific research: an educational project supported by a national society for clinical pharmacy." (2019): A285-A286. | Insufficient description of education intervention |
| Winzenberg T, Callisaya M, Hides J. Meeting the challenge of research capacity building: the Tasmanian Physiotherapy Research Group. Physiotherapy. 2015 May 1;101:e1651-2. | Insufficient description of education intervention |
| Scott, C.; Booth-LaForce, C.; Zwickey, H.; Bradley, R. Building research across interdisciplinary gaps clinical research training program in complementary and integrative health. Global Advances In Health and Medicine. 2018 | Insufficient description of education intervention |
| Rocco G, Affonso D, Mayberry L, Sasso L, Stievano A, Alvaro R. Center of Excellence to build nursing scholarship and improve health care in Italy. Journal of Nursing Scholarship. 2015 Mar;47(2):170-7. | Insufficient description of education intervention |
| Oancea, M.; Palmer, Z.; Wiseman, T.; Bell, C.; St Westwood, G. The value of investment in nursing leadership to build research capability and capacity in hospices and deliver high quality research within the multidisciplinary team. Palliative medicine. 2021; 35(1) Suppl. | Insufficient description of education intervention |
| O'Hearn, K.; Ward, R.; Foster, D.; Clayton, L.; Julien, L.; Marten, N.; Matte, A.; McDonald, E.; Smith, O.; Van Huyse, J.; Zytaruk, N.; Cook, D. Promoting professional development of critical care research coordinators through annual workshops. Canadian Journal of Anesthesia. 2019; 66(1) | Insufficient description of education intervention |
| Pereira, Rui Pedro Gomes, Maria José Peixoto, Teresa Martins, Maria do Céu Barbieri, and António Vaz Carneiro. An impact evaluation study of a short intensive course training in evidence-based nursing. (2016). | Insufficient description of education intervention |
| Dryden-Palmer K, Van Huyse J, Hutchison J. THE TERRY HICKSON RESEARCH IN PRACTICE CHALLENGE: INTEGRATING RESEARCH AND PRACTICE. Pediatric Critical Care Medicine. 2018;19(6). | Insufficient description of education intervention |
| Ploeg J, De Witt L, Hutchison B, Hayward L, Grayson K. Evaluation of a research mentorship program in community care. Evaluation and Program Planning. 2008 Feb 1;31(1):22-33. | Insufficient description of education intervention |
| Nightingale J, Fowler-Davis S, Grafton K, Kelly S, Langham C, Lewis R, Bianco B, Harrop D. The role of allied health professions and nursing research internships in developing a research culture: a mixed-methods exploration of stakeholder perspectives. Health Research Policy and Systems. 2020 Dec;18(1):1-7. | Insufficient description of education intervention |
| Sabey A, Bray I, Gray S. Building capacity to use and undertake applied health research: establishing a training programme for the health workforce in the West of England. public health. 2019 Feb 1;167:62-9. | Insufficient description of education intervention |
| Ried K, Farmer EA, Weston KM. Bursaries, writing grants and fellowships: a strategy to develop research capacity in primary health care. BMC Family Practice. 2007 Dec;8(1):1-3. | Insufficient description of education intervention |
| Miller C, Cook J, Gibson JM, Watkins CL, Jones SP. Clinical academic research internships for nurses, midwives and allied health professionals: a qualitative evaluation. Nurse Researcher. 2020 Sep 16;28(3). | Insufficient description of education intervention |
| Wallen GR, Mitchell SA, Melnyk B, Fineout‐Overholt E, Miller‐Davis C, Yates J, Hastings C. Implementing evidence‐based practice: effectiveness of a structured multifaceted mentorship programme. Journal of advanced nursing. 2010 Dec;66(12):2761-71. | Insufficient description of education intervention |
| Khan K, Porroche-Escudero A, Georgiou G, Popay J. Building Health Research Capacity: The Impact of a UK Collaborative Programme. Internet Journal of Allied Health Sciences and Practice. 2021 Sep 15;19(4). | Insufficient description of education intervention |
| Gottschlich, M. M.; Fowler, L. A.; Kagan, R. J. Journal club promotes clinical research, continuing education and evidence-based practice. Journal of Burn Care and Research. 2012 | Insufficient description of education intervention |
| Harding KE, Shields N, Whiteside M, Taylor NF. “A Great First Step into Research”: Stepping Into Research Is an Effective and Sustainable Model for Research Training in Clinical Settings A Report of 6-Year Outcomes. Journal of Allied Health. 2016 Sep 6;45(3):176-82. | Insufficient description of education intervention |
| Stutzman S, Olson D, Supnet C, Harper C, Brown-Cleere S, McCulley B, Goldberg M. Promoting bedside nurse-led research through a dedicated neuroscience nursing research fellowship. The Journal of Nursing Administration. 2016 Dec 1;46(12):648-53. | Insufficient description of education intervention |
| Lau F, Hayward R. Building a virtual network in a community health research training program. Journal of the American Medical Informatics Association. 2000 Jul 1;7(4):361-77. | Insufficient description of education intervention |
| Vachon B, Durand MJ, LeBlanc J. Empowering occupational therapists to become evidence-based work rehabilitation practitioners. Work. 2010 Jan 1;37(2):119-34. | Insufficient description of education intervention |
| Fullam J, Cusack E, Nugent LE. Research excellence across clinical healthcare: a novel research capacity building programme for nurses and midwives in a large Irish region. Journal of Research in Nursing. 2018 Dec;23(8):692-706. | Insufficient description of education intervention |
| Bryar RM. Practitioner research: An approach to developing research capacity in primary care. NT Research. 2003 Mar;8(2):101-14. | Insufficient description of education intervention |
| Carter EJ, Cato KD, Rivera RR, Kulage KM, Liu J, Vose C, Larson E. Programmatic details and outcomes of an Academic-Practice Research Fellowship for clinical nurses. Applied Nursing Research. 2020 Oct 1;55:151296. | Insufficient description of education intervention |
| Clancey JK. Nurse research internship program: A unique mentoring program. Journal of Neuroscience Nursing. 2009 Dec 1;41(6):E1-6. | Insufficient description of education intervention |
| Fischer-Cartlidge E. An Evidence-Based Approach to Increasing Nurses' Publication Rates. AJN The American Journal of Nursing. 2020 Aug 1;120(8):50-5. | Insufficient description of education intervention |
| Gawlinski A, Miller PS. Advancing nursing research through a mentorship program for staff nurses. AACN advanced critical care. 2011 Jul;22(3):190-200. | Insufficient description of education intervention |
| HAMMICK M. A research and journal club: a medium for teaching, professional development and networking. European Journal of Cancer Care. 1995 Mar;4(1):33-7. | Insufficient description of education intervention |
| Henry BJ, Bucher L, Mackley A, Eckman T. A novel approach to improving current research awareness: a Web-based Nursing Research Journal Watch. Journal for Nurses in Professional Development. 2010 Sep 1;26(5):208-14. | Insufficient description of education intervention |
| Kumar AM, Shewade HD, Tripathy JP, Guillerm N, Tayler-Smith K, Dar Berger S, Bissell K, Reid AJ, Zachariah R, Harries AD. Does research through Structured Operational Research and Training (SORT IT) courses impact policy and practice? Public Health Action. 2016 Mar 21;6(1):44-9. | Insufficient description of education intervention |
| Adams J, Kawchuk G, Breen A, De Carvalho D, Eklund A, Fernandez M, Funabashi M, Holmes MM, Johansson MS, de Luca K, Moore C. Leadership and capacity building in international chiropractic research: introducing the chiropractic academy for research leadership (CARL). Chiropractic & Manual Therapies. 2018 Dec;26(1):1-6. | Insufficient description of education intervention |
| Linde BJ. The effectiveness of three interventions to increase research utilization among practicing nurses (Doctoral dissertation, University of Michigan). | Insufficient description of education intervention |
| Webster E, Thomas M, Ong N, Cutler L. Rural research capacity building program: capacity building outcomes. Australian Journal of Primary Health. 2011 Apr 6;17(1):107-13 | Insufficient description of education intervention |
| Impact case studies [Impact of a Research Capacity Building Initiative: Qualitative Evaluation of HP Research Positions (health.qld.gov.au)](https://www.health.qld.gov.au/__data/assets/pdf_file/0025/503773/qerpart2-casestudies.pdf) (grey literature) | Insufficient description of education intervention |
| Annual Research Report, Education and Research Support  [2019 Annual Research Report - Research and Innovation within the Sunshine Coast Hospital and Health Service](https://www.health.qld.gov.au/__data/assets/pdf_file/0018/1011429/2019-annual-research-report.pdf) (grey literature) | Insufficient description of education intervention |
| Research, teaching and training  [ACT Health Summit: Research, Teaching and Training \| Health](https://www.health.act.gov.au/research/act-health-summit-research-teaching-and-training) (grey literature) | Insufficient description of education intervention |
| Development and Research  [Development and Research \| Careers (health.tas.gov.au)](https://www.health.tas.gov.au/career/home/nurses2/development_and_research) (grey literature) | Insufficient description of education intervention |
| [Health research - NT Health](https://health.nt.gov.au/data-and-research/nt-health-research) (grey literature) | Insufficient description of education intervention |
| [Designing](https://sarrah.org.au/cpd/online-education/sarrah-practice-redesign-series?highlight=WyJyZXNlYXJjaCIsImVkdWNhdGlvbiJd) and implementing successful AHA modes of care  [Designing and Implementing Successful AHA Models of Care - SARRAH](https://sarrah.org.au/cpd/online-education/online-courses/521-designing-and-implementing-successful-aha-models-of-care?highlight=WyJyZXNlYXJjaCIsImVkdWNhdGlvbiJd) | Insufficient description of education intervention |
| Impact of a research capacity building initiative  [Impact of a Research Capacity Building Initiative: Qualitative Evaluation of HP Research Positions (health.qld.gov.au)](https://www.health.qld.gov.au/__data/assets/pdf_file/0032/498380/qerpart1-qualeval.pdf) | Insufficient description of education intervention |
| Lobo R, Crawford G, Hallett J, Laing S, Mak DB, Jancey J, Rowell S, McCausland K, Bastian L, Sorenson A, Tilley PM. A research and evaluation capacity building model in Western Australia. Health Promotion International. 2018 Jun 1;33(3):468-78. | No research education intervention was described |
| Harne-Britner S, Schafer DJ. Clinical nurse specialists driving research and practice through research roundtables. Clinical Nurse Specialist. 2009 Nov 1;23(6):305-8. | No research education intervention was described |
| Goodenough B, Fleming R, Young M, Burns K, Jones C, Forbes F. Raising awareness of research evidence among health professionals delivering dementia care: Are knowledge translation workshops useful?. Gerontology & geriatrics education. 2017 Oct 2;38(4):392-406. | No research education intervention was described |
| Dall'Oglio I, Vanzi V, Tiozzo E, Gawronski O, Biagioli V, Tucci S, Raponi M. Five years of journal clubs with pediatric nurses and allied health professionals: A retrospective study and satisfaction survey. Journal of Pediatric Nursing. 2018 Jul 1;41:e2-7. | No research education intervention was described |
| Kjerholt M, Hølge‐Hazelton B. Cultivating a culture of research in nursing through a journal club for leaders: A pilot study. Journal of Nursing Management. 2018 Jan;26(1):42-9. | No research education intervention was described |
| Cooke J, Nancarrow S, Dyas J, Williams M. An evaluation of the'Designated Research Team'approach to building research capacity in primary care. BMC Family Practice. 2008 Dec;9(1):1-2. | No research education intervention was described |
| Bell CA, Colleran V. Empowering nurses, midwives and allied health professionals to gain an academic, research and quality improvement experience within clinical practice. International Journal of Practice-Based Learning in Health and Social Care. 2019 Dec 13;7(2):69-79. | No research education intervention was described |
| Janssen J, Hale L, Mirfin-Veitch B, Harland T. Building the research capacity of clinical physical therapists using a participatory action research approach. Physical therapy. 2013 Jul 1;93(7):923-34. | No research education intervention was described |
| Stone T, Levett-Jones T, Harris M, Sinclair PM. The genesis of ‘the Neophytes’: A writing support group for clinical nurses. Nurse Education Today. 2010 Oct 1;30(7):657-61. | No research education intervention was described |
| Duffy JR, Culp S, Yarberry C, Stroupe L, Sand-Jecklin K, Coburn AS. Nurses’ research capacity and use of evidence in acute care: Baseline findings from a partnership study. JONA: The Journal of Nursing Administration. 2015 Mar 1;45(3):158-64. | No research education intervention was described |
| Bell E, Davies L, Squibb K, Cannell R. Research capacity-building in primary health care: What works?. Public Health Yearbook. 2011. 2011:471. | No research education intervention was described |
| Kennedy AB. Journal aspirations: Improving scientific writing and publication through a writing mentorship program. International journal of therapeutic massage & bodywork. 2017 Jun;10(2):1. | No research education intervention was described |
| Foran-Tuller K, Robiner WN, Breland-Noble A, Otey-Scott S, Wryobeck J, King C, Sanders K. Early career boot camp: A novel mechanism for enhancing early career development for psychologists in academic healthcare. Journal of Clinical Psychology in Medical Settings. 2012 Mar;19(1):117-25. | No research education intervention was described |
| McCreesh K, Larkin L, Lewis J. Shouldering the burden of evidence-based practice: the experiences of physiotherapists partaking in a Community of Practice. Rehabilitation Research and Practice. 2016 Jan 24;2016. | No research education intervention was described |
| McNeill T, Nicholas DB. Strategies for research development in hospital social work: A case study. Research on Social Work Practice. 2012 Nov;22(6):672-9. | No research education intervention was described |
| Hauck YL, Lewis L, Bayes S, Keyes L. Research capacity building in midwifery: case study of an Australian graduate midwifery research intern programme. Women and Birth. 2015 Sep 1;28(3):259-63. | No research education intervention was described |
| Hogg W, Donskov M, Russell G, Pottie K, Liddy C, Johnston S, Chambers L. Riding the wave of primary care research: Development of a primary health care research centre. Canadian Family Physician. 2009 Oct 1;55(10):e35-40. | No research education intervention was described |
| Nicholson K, Ganann R, Bookey-Bassett S, Baird LG, Garnett A, Marshall Z, Khan AI, Pirrie M, Sasseville M, Charif AB, Poitras MÈ. Capacity building and mentorship among pan-Canadian early career researchers in community-based primary health care. Primary health care research & development. 2020;21. | No research education intervention was described |
| Dorgan S. Building research capacity and capability in the nursing, midwifery and allied health professions. British Journal of Nursing (Mark Allen Publishing). 2018 Jun 1;27(11):634-5. | No research education intervention was described |
| Parker V, Lieschke G, Giles M. Ground-up-top down: a mixed method action research study aimed at normalising research in practice for nurses and midwives. BMC nursing. 2017 Dec;16(1):1-8. | No research education intervention was described |
| Ried K, Fuller J. Building a culture of research dissemination in primary health care: the South Australian experience of supporting the novice researcher. Australian Health Review. 2005;29(1):6-11. | No research education intervention was described |
| Crozier K, Moore J, Kite K. Innovations and action research to develop research skills for nursing and midwifery practice: the Innovations in Nursing and Midwifery Practice Project study. Journal of Clinical Nursing. 2012 Jun;21(11‐12):1716-25. | No research education intervention was described |
| Ward EC, Hargrave C, Brown E, Halkett G, Hogg P. Achieving success in clinically based research: the importance of mentoring. Journal of medical radiation sciences. 2017 Dec;64(4):315-20. | No research education intervention was described |
| Likumahuwa S, Song H, Singal R, Weir RC, Crane H, Muench J, Sim SC, DeVoe JE. Building research infrastructure in community health centers: a Community Health Applied Research Network (CHARN) report. The Journal of the American Board of Family Medicine. 2013 Sep 1;26(5):579-87. | No research education intervention was described |
| Ruco A, Nichol K, Morassaei S, Bola R, Di Prospero L. Supporting Discovery and Inquiry: A Canadian Hospital's Approach to Building Research and Innovation Capacity in Point-of-Care Health Professionals. Quality Management in Health Care. 2021 Oct 28;30(4):267-75. | No research education intervention was described |
| Bell T, Vat LE, McGavin C, Keller M, Getchell L, Rychtera A, Fernandez N. Co-building a patient-oriented research curriculum in Canada. Research involvement and engagement. 2019 Dec;5(1):1-3. | No research education intervention was described |
| Payne S, Seymour J, Grande G, Froggatt K, Molassiotis A, Lloyd-Williams M, Foster C, Addington-Hall J, Rolls E, Todd C. An evaluation of research capacity building from the Cancer Experiences Collaborative. BMJ supportive & palliative care. 2012 Sep 1;2(3):280-5. | No research education intervention was described |
| Bennett S, Whitehead M, Eames S, Fleming J, Low S, Caldwell E. Building capacity for knowledge translation in occupational therapy: learning through participatory action research. BMC medical education. 2016 Dec;16(1):1-1. | No research education intervention was described |
| Barbour RS, Featherstone VA. Acquiring qualitative skills for primary care research. Review and reflections on a three-stage workshop. Part 1: using interviews to generate data. Family Practice. 2000 Feb 1;17(1):76-82. | Does not evaluate research program |
| Barbour RS, Featherstone VA. Acquiring qualitative skills for primary care research. Review and reflections on a three-stage workshop. Part 1: using interviews to generate data. Family Practice. 2000 Feb 1;17(1):76-82. | Does not evaluate research program |
| Rigau BL, Dooling-Litfin J, Scully E, Killian C, Fisher G, McManus B, Khetani MA. Building Organizational Capacity for Research in Early Intervention. ZERO TO THREE. 2019 Jul;39(6):54-61. | Does not evaluate research program |
| Crausaz J, Kelly M, Lee S. Three educational approaches to enhance the evidence-based practice behaviour of Irish occupational therapists. World Federation of Occupational Therapists Bulletin. 2011 Nov 1;64(1):11-7. | Does not evaluate research program |
| Dowding D. Building research capacity and capability in nursing, midwifery and allied health professionals in Scotland. Report to SHEFC and SFC. 2003. | Does not evaluate research program |
| Aloweni F, Teh AH, Tan SB, Ang SY. Promoting research competence: introduction of the nursing research immersion program in Singapore General Hospital. Proceedings of Singapore Healthcare. 2017 Dec;26(4):267-9. | Does not evaluate research program |
| Carthon JM, Holland S, Gamble K, Rothwell H, Pancir D, Ballinghoff J, Aiken L. Increasing research capacity in a safety net setting through an academic clinical partnership. JONA: The Journal of Nursing Administration. 2017 Jun 1;47(6):350-5. | Does not evaluate research program |
| Cooper S, Sanders J, Pashayan N. Implementing a novel programme for nurses and allied health professionals to develop capacity for evidence-informed clinical practice. Journal of Research in Nursing. 2021 Aug;26(5):395-404. | Does not evaluate research program |
| Chan RJ, Bowers A, Barton-Burke M. Organizational strategies for building capacity in evidence-based oncology nursing practice: a case report of an Australian tertiary cancer center. Nursing Clinics. 2017 Mar 1;52(1):149-58. | Does not evaluate research program |
| Ghebrehewet S, Harries AD, Kliner M, Smith K, Cleary P, Wilkinson E, Stewart A. Adapting the Structured Operational Research Training Initiative (SORT IT) for high-income countries. Public Health Action. 2019 Jun 21;9(2):69-71. | Does not evaluate research program |
| Laizner AM. A research course to promote evidence-based nursing practice. Canadian Oncology Nursing Journal= Revue Canadienne de Nursing Oncologique. 2005 Jan 1;15(4):256-7. | Does not evaluate research program |
| McGuire DB, Walczak JR, Krumm SL. Development of a nursing research utilization program in a clinical oncology setting: organization, implementation, and evaluation. InOncology nursing forum 1994 May 1 (Vol. 21, No. 4, pp. 704-710). | Does not evaluate research program |
| Doswell W, Smith AM. The nursing research internship program: providing research training in the clinical setting. The Journal of the New York State Nurses' Association. 1993 Sep 1;24(3):11-4. | Does not evaluate research program |
| Bailey J, Veitch C, Crossland L, Preston R. Developing research capacity building for Aboriginal and Torres Strait Islander health workers in health service settings. Rural and remote health. 2006 Dec 1;6(4):1-0. | Does not evaluate research program |
| Burridge L, New A, Lewis D, Kohlhardt J, Tattam T. Nurse-led research capacity building: Developing a local strategy with direct-care rehabilitation nurses. Journal of the Australasian Rehabilitation Nurses Association. 2020 Dec;23(2):17-24. | Does not evaluate research program |
| Burt T, Sharma P, Mittal S. Research question, study design and continuous research education and training exercises (CREATE) program. Journal of clinical and preventive cardiology. 2012 Jan;1(1):35. | Does not evaluate research program |
| Umlauf MG, Sherman S. Facilitating research utilization through collaboration. Nursing Connections. 1992 Jan 1;5(2):37-42. | Does not evaluate research program |
| Rural Research Capacity Building Program Information and Application Guidelines  <https://www.heti.nsw.gov.au/__data/assets/pdf_file/0003/671817/RRCBP-Information-and-Application-Guidelines-2022.pdf> (grey literature) | Does not evaluate research program |
| RETProgram <https://wahtn.org/platforms/research-education-training-program/> (grey literature) | Does not evaluate research program |
| Training and Education (Allied Health Translating Research into Practice) [Training and education \| Queensland Health](https://www.health.qld.gov.au/clinical-practice/database-tools/translating-research-into-practice-trip/translating-research-into-practice) (grey literature) | Does not evaluate research program |
| Rural Research Capacity Building Program Information and Application Guidelines  <https://www.heti.nsw.gov.au/__data/assets/pdf_file/0003/671817/RRCBP-Information-and-Application-Guidelines-2022.pdf> (grey literature) | Does not evaluate research program |
| RETProgram <https://wahtn.org/platforms/research-education-training-program/> (grey literature) | Does not evaluate research program |
| <https://www.seslhd.health.nsw.gov.au/services-clinics/directory/research-home/research-education> | Does not evaluate research program |
| Carlisle C, Ibbotson T. Introducing problem-based learning into research methods teaching: Student and facilitator evaluation. Nurse education today. 2005 Oct 1;25(7):527-41. | Part of tertiary education |
| Lacey EA. Facilitating research-basedpractice by educational intervention. Nurse Education Today. 1996 Aug 1;16(4):296-301. | Part of tertiary education |
| Begley C, McCarron M, Huntley-Moore S, Condell S, Higgins A. Successful research capacity building in academic nursing and midwifery in Ireland: An exemplar. Nurse Education Today. 2014 May 1;34(5):754-60. | Part of tertiary education |
| Chilton J, He Z, Fountain R, Alfred D. A process for teaching research methods in a virtual environment. Journal of Professional Nursing. 2019 Mar 1;35(2):101-4. | Part of tertiary education |
| Edwards N, Smith D. Building research capacity: an internship program puts community nursing researchers on the fast track for developing skills essential to successfully apply for grants, linking with decision-makers and other researchers, and exploring new approaches to research. The Canadian Nurse. 2003 Oct 1;99(9):23. | Part of tertiary education |
| Hazel R, Joyce A. The long-term effects of undertaking a research course on clinical practice. Nurse Education in Practice. 2004 Mar 1;4(1):12-9. | Part of tertiary education |
| Cheek J, Gillham D, Ballantyne A. Using education to promote research dissemination in nursing. International Journal of Nursing Education Scholarship. 2005 Dec 9;2(1). | Part of tertiary education |
| Kumar S, Perraton L, Machotka Z. Development and implementation of an online hybrid model for teaching evidence-based practice to health professions: processes and outcomes from an Australian experience. Advances in Medical Education and Practice. 2010;1:1. | Part of tertiary education |
| Morrison-Beedy D, Côté-Arsenault D. The cookie experiment revisited: broadened dimensions for teaching nursing research. Nurse Educator. 2000 Nov 1;25(6):294-6. | Part of tertiary education |
| Murrock CJ. Innovative short group writing assignments to enhance scholarly writing skills. Journal of Nursing Education. 2019;58(1):61-. | Part of tertiary education |
| Zhang Q, Shi Y, Xin Y, Zhang S, Zeng N, Liu M, Wu S, Wei W, Li M, You H, Jia J. A multimodal international collaborative clinical research training program in China. Medical education online. 2019 Jan 1;24(1):1679944. | Part of tertiary education |
| Petracchi HE, Patchner ME. A comparison of live instruction and interactive televised teaching: A 2-year assessment of teaching an MSW research methods course. Research on Social Work Practice. 2001 Jan;11(1):108-17. | Part of tertiary education |
| Rhondali W, Nguyen LMT, Peck M, Vallet F, Daneault S, Filbet M. Description of a teaching method for research education for palliative care healthcare professionals. Palliat Support Care. 2015;13(2):249-54. | Not delivered in healthcare setting/context (e.g., the higher education setting) |
| Baldwin JA, Williamson HJ, Eaves ER, Levin BL, Burton DL, Massey OT. Broadening measures of success: results of a behavioral health translational research training program. Implementation Science. 2017 Dec;12(1):1-1. | Not delivered in healthcare setting/context (e.g., the higher education setting) |
| Bäck‐Pettersson S, Jensen KP, Kylén S, Sernert N, Hermansson E. Nurses' experiences of participation in a research and development programme. Journal of Clinical Nursing. 2013 Apr;22(7-8):1103-11. | Not delivered in healthcare setting/context (e.g., the higher education setting) |
| Breckenridge-Sproat ST, Throop MD, Raju D, Murphy DA, Loan LA, Patrician PA. Building a unit-level mentored program to sustain a culture of inquiry for evidence-based practice. Clinical Nurse Specialist. 2015 Nov 1;29(6):329-37. | Not delivered in healthcare setting/context (e.g., the higher education setting) |
| Jackson D. Mentored residential writing retreats: A leadership strategy to develop skills and generate outcomes in writing for publication. Nurse education today. 2009 Jan 1;29(1):9-15. | Not delivered in healthcare setting/context (e.g., the higher education setting) |
| Norton WE, Zwarenstein M, Czajkowski S, Kato E, O’Mara A, Shelburne N, Chambers DA, Loudon K. Building internal capacity in pragmatic trials: a workshop for program scientists at the US National Cancer Institute. Trials. 2019 Dec;20(1):1-6. | Not delivered in healthcare setting/context (e.g., the higher education setting) |
| Zick SM, Benn R. Bridging CAM practice and research: teaching CAM practitioners about research methodology. Alternative Therapies in Health & Medicine. 2004 May 1;10(3). | Not delivered in healthcare setting/context (e.g., the higher education setting) |
| Stajic J, Harfield S, Brown A, Dawson A, Davy C, Aromataris E, Braunack-Mayer A. Evaluating a research capacity strengthening program for Aboriginal community-controlled health organisations. Australian Journal of Primary Health. 2019 Mar 5;25(1):72-81. | Not delivered in healthcare setting/context (e.g., the higher education setting) |
| Perry LA. The Bridge Program: an overview. The ABNF Journal: Official Journal of the Association of Black Nursing Faculty in Higher Education, Inc. 1997 Jan 1;8(1):4-7. | Not delivered in healthcare setting/context (e.g., the higher education setting) |
| Ponte PR, Hayman LL, Berry DL, Cooley ME. A new model for postdoctoral training: The Nursing Postdoctoral Program in Cancer and Health Disparities. Nursing outlook. 2015 Mar 1;63(2):189-203. | Not delivered in healthcare setting/context (e.g., the higher education setting) |
| Shrivastava M, Shah N, Navaid S. Assessment of change in knowledge about research methods among delegates attending research methodology workshop. Perspectives in clinical research. 2018 Apr;9(2):83. | Program delivered in low- or middle-income country |
| Tsai SL. The effects of a research utilization in-service program on nurses. International journal of nursing studies. 2003 Feb 1;40(2):105-13. | Program delivered in low- or middle-income country |
| Bates I. Designing and measuring the progress and impact of health research capacity strengthening initiatives. In BMC proceedings 2015 Dec (Vol. 9, No. 10, pp. 1-6). BioMed Central. | Program delivered in low- or middle-income country |
| Sridharan S, Bondy M, Nakaima A, Heller RF. The potential of an online educational platform to contribute to achieving sustainable development goals: a mixed-methods evaluation of the Peoples-uni online platform. Health Research Policy and Systems. 2018 Dec;16(1):1-4. | Program delivered in low- or middle-income country |
| Rose R, Das H, Narayan J, Jament J. Training in Qualitative Research Methods for Professionals working with Persons with Disabilities. Disability, CBR and Inclusive Development. 2021 May 6;32(1):130-49. | Program delivered in low- or middle-income country |
| Aston, M., Dumont, C., Edwards, S., et al. Innovative nursing research internship. Canadian Nurse. 2003; 99(1). | Full text inaccessible |
| [CAHPR Cheshire & Merseyside event - First Steps in Research \| Council for Allied Health Professions Research (csp.org.uk)](https://cahpr.csp.org.uk/news/2018-03-01-cahpr-cheshire-merseyside-event-first-steps-research) | Full text inaccessible |
| Perry LI, Grange A, Heyman BO, Noble P. Stakeholders’ perceptions of a research capacity development project for nurses, midwives and allied health professionals. Journal of Nursing Management. 2008 Apr;16(3):315-26. | Described new role (e.g., librarian, knowledge broker) as intervention |
| Davis III TH, Wagner GS, Gleim G, Andolsek KM, Arheden H, Austin R, Courtney-Eighmy A, Gradison M, Leist JC, Maynard C, Noga Jr EM. Problem-based learning of research skills. Journal of Electrocardiology. 2006 Jan 1;39(1):120-8. | Targeted at medical doctors only |
